# Genotypic Diversity within a Single *Pseudomonas aeruginosa* Strain Commonly Shared by Australian Patients with Cystic Fibrosis

**DOI:** 10.1371/journal.pone.0144022

**Published:** 2015-12-03

**Authors:** Anna Sze Tai, Scott Cameron Bell, Timothy James Kidd, Ella Trembizki, Cameron Buckley, Kay Annette Ramsay, Michael David, Claire Elizabeth Wainwright, Keith Grimwood, David Mark Whiley

**Affiliations:** 1 Queensland Children’s Medical Research Institute, Children’s Health Queensland, South Brisbane, Queensland, Australia; 2 Adult Cystic Fibrosis Centre, Department of Thoracic Medicine, The Prince Charles Hospital, Chermside, Queensland, Australia; 3 School of Medicine, The University of Queensland, Herston, Queensland, Australia; 4 QIMR Berghofer Medical Research Institute, Herston, Queensland, Australia; 5 Centre for Infection and Immunity, Queen’s University Belfast, Belfast, United Kingdom; 6 School of Population Health, The University of Queensland, Herston, Queensland, Australia; 7 Department of Respiratory and Sleep Medicine, Lady Cilento Children’s Hospital, South Brisbane, Queensland, Australia; 8 Menzies Health Institute Queensland, Griffith University and the Gold Coast University Hospital, Gold Coast, Queensland, Australia; 9 Pathology Queensland Central Laboratory, Herston, Queensland, Australia; Lee Kong Chian School of Medicine, SINGAPORE

## Abstract

In cystic fibrosis (CF), *Pseudomonas aeruginosa* undergoes intra-strain genotypic and phenotypic diversification while establishing and maintaining chronic lung infections. As the clinical significance of these changes is uncertain, we investigated intra-strain diversity in commonly shared strains from CF patients to determine if specific gene mutations were associated with increased antibiotic resistance and worse clinical outcomes. Two-hundred-and-one *P*. *aeruginosa* isolates (163 represented a dominant Australian shared strain, AUST-02) from two Queensland CF centres over two distinct time-periods (2001–2002 and 2007–2009) underwent *mexZ* and *lasR* sequencing. Broth microdilution antibiotic susceptibility testing in a subset of isolates was also performed. We identified a novel AUST-02 subtype (M3L7) in adults attending a single Queensland CF centre. This M3L7 subtype was multi-drug resistant and had significantly higher antibiotic minimum inhibitory concentrations than other AUST-02 subtypes. Prospective molecular surveillance using polymerase chain reaction assays determined the prevalence of the ‘M3L7’ subtype at this centre during 2007–2009 (170 patients) and 2011 (173 patients). Three-year clinical outcomes of patients harbouring different strains and subtypes were compared. *MexZ* and LasR sequences from AUST-02 isolates were more likely in 2007–2009 than 2001–2002 to exhibit mutations (*mexZ*: odds ratio (OR) = 3.8; 95% confidence interval (CI): 1.1–13.5 and LasR: OR = 2.5; 95%CI: 1.3–5.0). Surveillance at the adult centre in 2007–2009 identified M3L7 in 28/509 (5.5%) *P*. *aeruginosa* isolates from 13/170 (7.6%) patients. A repeat survey in 2011 identified M3L7 in 21/519 (4.0%) *P*. *aeruginosa* isolates from 11/173 (6.4%) patients. The M3L7 subtype was associated with greater intravenous antibiotic and hospitalisation requirements, and a higher 3-year risk of death/lung transplantation, than other AUST-02 subtypes (adjusted hazard ratio [HR] = 9.4; 95%CI: 2.2–39.2) and non-AUST-02 strains (adjusted HR = 4.8; 95%CI: 1.4–16.2). This suggests ongoing microevolution of the shared CF strain, AUST-02, was associated with an emerging multi-drug resistant subtype and possibly poorer clinical outcomes.

## Introduction

Initial pulmonary infections with *Pseudomonas aeruginosa* in patients with cystic fibrosis (CF) are typically intermittent and caused by non-mucoid environmental strains that are susceptible to anti-pseudomonal antibiotics [1–3]. Eventually, a single strain becomes established and develops a mucoid phenotype, which with increasing antibiotic resistance makes eradication from the airways difficult[4]. Once this event occurs, patients experience an accelerated pulmonary decline with increased exacerbation frequency and mortality [5].

While environmental origins of *P*. *aeruginosa* in CF patients are well recognised, reports of patients sharing indistinguishable strains suggest cross-infection as another acquisition pathway [6, 7]. Importantly, shared CF strains can have different phenotypic properties and clinical impact. In 2004 the Liverpool Epidemic strain (LES) accounted for 11% of *P*. *aeruginosa* infected patients attending 15 surveyed CF clinics in England and Wales [8]. Compared with other *P*. *aeruginosa* strains, it had increased antibiotic resistance and was associated with greater declines in lung function and nutritional status [9, 10]. LES was also found in 15% of patients attending CF clinics in Ontario, Canada where it was associated with an increased risk of death or lung transplantation [11]. Similarly, the Manchester Epidemic Strain, detected in 14% of patients attending a Manchester CF clinic was multi-drug resistant (MDR) and associated with increased antibiotic and healthcare use, but not mortality [12]. In Australia, a national microbiological survey of CF centres found two strains, AUST-01 and AUST-02, in 22% and 18% of CF patients harbouring *P*. *aeruginosa* respectively [13]. Furthermore, regional differences existed and AUST-02 was observed in 35%-40% of infected patients attending Queensland CF clinics [13, 14]. This strain had increased carbapenem and aminoglycoside antibiotic resistance and was associated with greater treatment requirements than unique strains [13, 15]. However, the actual long-term clinical impact of AUST-02 remains uncertain.

In addition to between-strain differences, extensive intra-strain diversity is observed in *P*. *aeruginosa* isolates from chronically infected CF airways [16–20]. The CF lung is a spatially heterogeneous structure resulting in differential exposures to antibiotics, host defences, oxidative stress, nutrition and oxygen availability. Consequently, the resident *P*. *aeruginosa* population demonstrates extensive *in-vivo* intra-strain genotypic and phenotypic diversity (or adaptive radiation) involving antibiotic susceptibility, colonial morphology, motility, biofilm formation, auxotrophy, quorum sensing and virulence factor expression during the course of chronic infection [16–20]. Genes encoding key regulatory networks, as well as metabolism, antibiotic resistance and virulence factors are particularly prone to mutations during chronic CF airway infection and the key regulatory genes for multi-drug efflux pumps (e.g. *mexZ*) and virulence factors (e.g. *lasR*) are among the most common mutational targets [16, 21, 22] *mexZ* inhibits the MexXY-OprM efflux pump, which is over-expressed when this gene is inactivated resulting in *P*. *aeruginosa* becoming less susceptible to aminoglycoside, β-lactam and fluoroquinolone antibiotics [23]. In contrast, inactivation of *lasR* decreases the expression of several virulence genes and confers a growth advantage in the amino acid enriched mucous secretions of the CF airway [24], while harbouring *P*. *aeruginosa* with *lasR* mutations is associated with accelerated progression of CF lung disease [25]. Nevertheless, whether these mutations affect transmissibility, morbidity and mortality of shared *P*. *aeruginosa* strains remains unclear. In this study, we used *mexZ* and *lasR* DNA sequencing to: (i) investigate intra-strain diversity within *P*. *aeruginosa* isolates from Queensland CF patients (involving mostly the commonly shared AUST-02 strain) and (ii) determine if mutations in these genes are associated with increased antibiotic resistance and poorer clinical outcomes.

## Methods

### Patient isolates and setting

**Fig 1:**
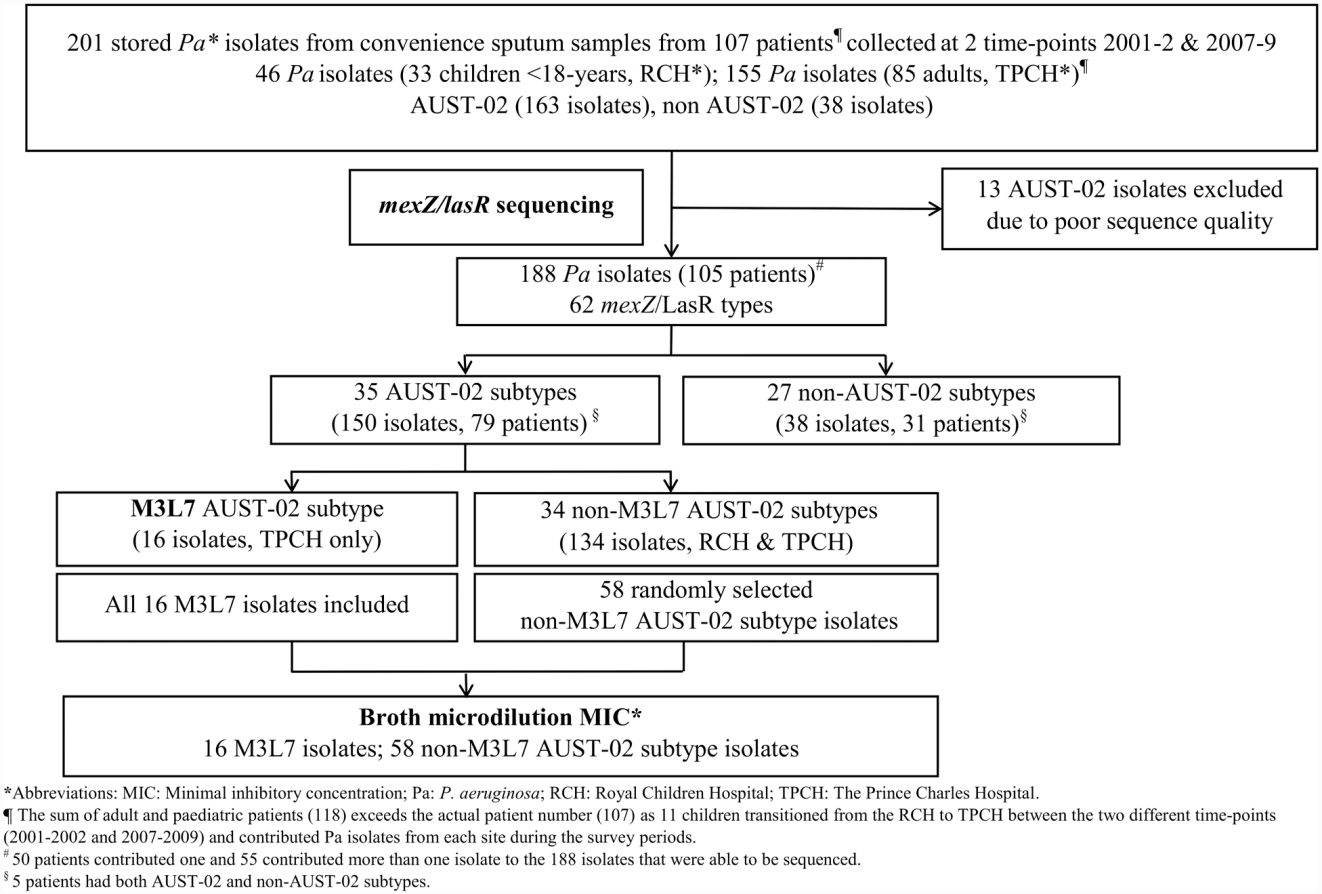
Flow diagram demonstrating patient and isolate selection for initial assessment of *mexZ* and *lasR* sequence diversity and antibiotic susceptibility.

**Fig 2:**
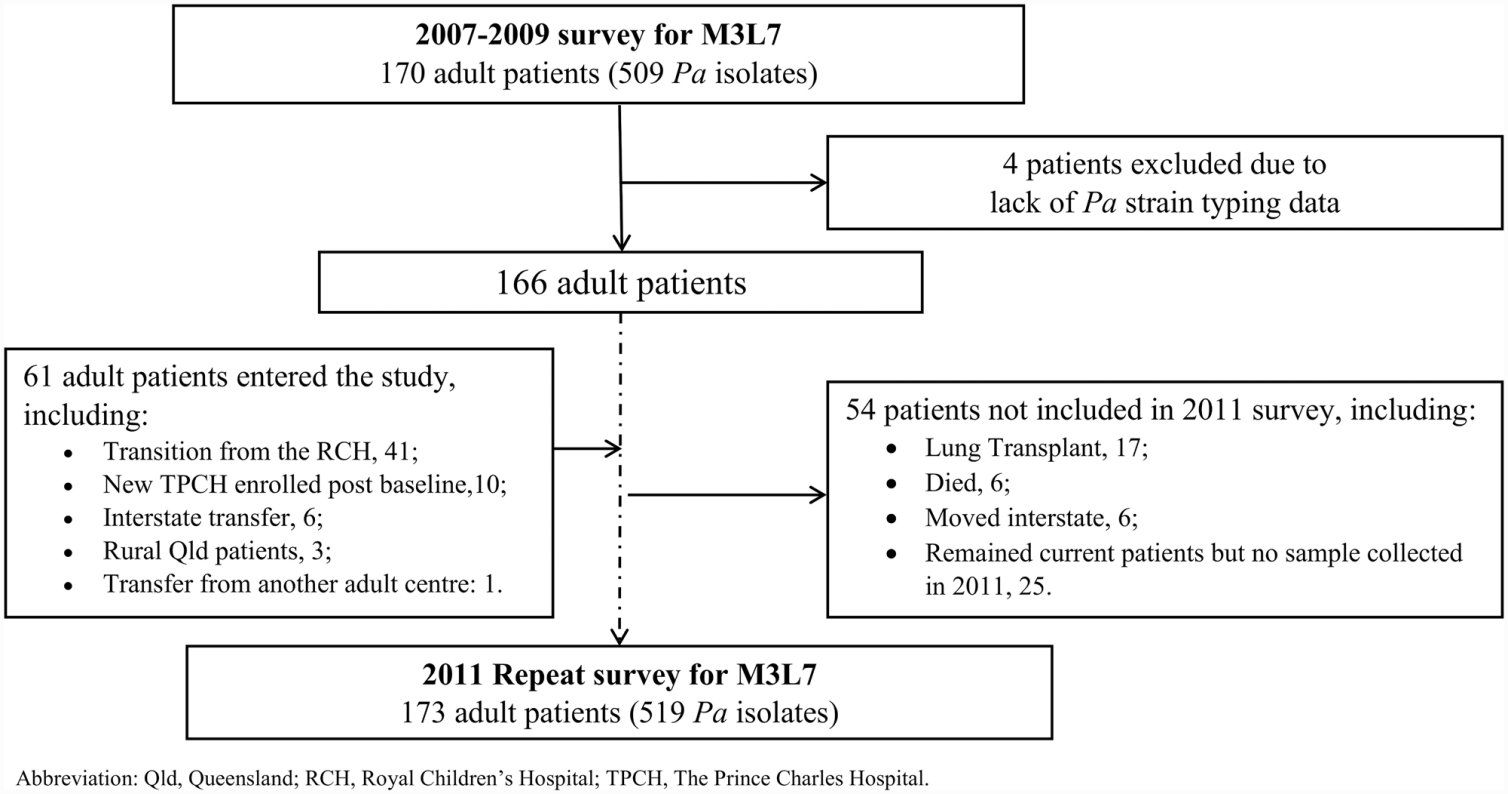
Flow diagram showing patients included in the two cross-sectional surveys for ‘M3L7’ AUST-02 subtype at TPCH.

This study, outlined in Figs 1 and 2, utilised stored *P*. *aeruginosa* isolates from two previous cross-sectional surveys [13, 15, 26], as well as the ongoing longitudinal Australian Clonal *P*. *aeruginosa* in Cystic Fibrosis (ACPinCF) study [13], which is linked with the Australian CF Data Registry (ACFDR). The Prince Charles Hospital, Children's Health Queensland and The University of Queensland Human Research Ethics Committees each approved the study. All participants (or their guardian for participants under 18-years) provided written informed consent on ethics committee approved participant consent forms.

### Initial assessment of *mexZ* and *lasR* sequence diversity and antibiotic susceptibility: 2001–2002 and 2007–2009

To assess intra-strain diversity within the *mexZ* and *lasR* gene sequences of *P*. *aeruginosa* strains, 201 stored *P*. *aeruginosa* isolates were assessed for *mexZ* and *lasR* sequence diversity. These isolates had been collected previously from 107 Queensland patients with CF attending either the Royal Children’s Hospital (RCH) or The Prince Charles Hospital (TPCH), Brisbane, Australia and were part of microbiological surveys conducted in 2001–2002 and 2007–2009 respectively (Fig 1) [13, 15, 26]. The isolates originated from sputum samples and had already been genotyped by enterobacterial repetitive intergenic consensus-polymerase chain reaction (ERIC-PCR) and Sequenom iPlex single nucleotide polymorphism (SNP)-based methods in earlier point-prevalence and typing studies [13, 26]. They included 163 AUST-02 isolates and 38 other minor shared and unique strains (S1 Table). *mexZ* and *lasR* gene PCR assays were performed using primers displayed in the S2 Table. Automated DNA sequencing of the amplicons was undertaken by The Australian Genome Research Facility using Big Dye Terminator chemistry version 3.1 (Applied Biosystems) and an Applied Biosystems 3730 capillary sequencer. Sequencing data were used to assign a subtype based on a *mexZ* intergenic and partial coding nucleotide sequence (approximately 755 bases) and the complete LasR protein (240 amino acids).

As outlined in Fig 1, the minimum inhibitory concentrations (MIC) for a subset of isolates to meropenem, imipenem, ceftazidime, ticarcillin-clavulanic acid, aztreonam, ciprofloxacin, tobramycin and colistin sulphate were determined by broth microdilution testing following the Clinical and Laboratory Standards Institute (CLSI) 2013 guidelines [27]. MDR was defined as resistance to all agents tested within two or more antibiotic groups, including the aminoglycosides, β-lactam agents and the fluoroquinolone, ciprofloxacin, according to the CF Foundation consensus guidelines [28, 29].

### Prevalence and clinical impact of ‘M3L7’ AUST-02 subtype in a single CF centre: 2007–2009 and 2011

Initial testing identified a novel AUST-02 *mexZ*/LasR subtype, ‘M3L7’, clustering in adult patients attending TPCH in 2007–2009. As this centre was participating in the longitudinal component of the ACPinCF study [13], patients were already providing one-to-three *P*. *aeruginosa* isolates from single sputum specimens obtained annually for culture. It was therefore possible to screen systematically for this M3L7 subtype in 509 *P*. *aeruginosa* isolates prospectively collected from the 170 infected adult CF patients attending this clinic between 2007–2009 and another 519 isolates from 173 patients attending the same clinic during 2011 (Fig 2).

‘M3L7’ detection was by SYBR-green based allele-specific real-time PCR assay (M3-PCR) targeting a SNP specific to the ‘M3’ *mexZ* sequence (primers detailed in the S2 Table). Isolates positive by M3-PCR had *lasR* DNA sequencing to confirm an M3L7 genotype. These M3L7 isolates underwent strain typing using a validated AUST-02 allele specific PCR assay (S2 Table).

### Clinical data collection

To assess the clinical impact of the M3L7 subtype, patients with *P*. *aeruginosa* identified in the 2007–2009 microbiological survey were followed longitudinally for up to 4-years from the time of their baseline sample collection until the census date of January 1^st^, 2011. Baseline clinical variables, including age, gender, CF genotype, pancreatic insufficiency, CF-related diabetes and/or liver disease, body-mass-index (BMI) at the time of the recorded forced expiratory volume in the first second (FEV_1_ [% predicted]) in the calendar year of sputum collection, the aforementioned FEV_1_% predicted, co-pathogens (*Burkholderia cepacia* complex, *Staphylococcus aureus*, *Achromobacter* and *Aspergillus* species), maintenance treatment (azithromycin, inhaled antibiotics and/or mucolytic therapies), as well as treatment episodes requiring intravenous (IV) antibiotics (number of episodes and days of hospitalisation) and the number of outpatient visits in the 12-months before sputum collection were recorded as described previously [13]. Longitudinal clinical data from individual patients were retrieved from the ACFDR for BMI, lung function measurements, frequency of hospitalisations and outpatient clinic visits, from the time of baseline isolate collection to the end of the study, death or lung transplantation, whichever came sooner.

### Statistical analyses

Clinical variables and MIC results were summarised using descriptive statistics. Continuous variables were expressed as either mean (standard deviation, SD) or median (interquartile range, IQR) values. Normally distributed data were compared using paired t-test (2 groups) and analysis of variance (>2 groups) with post-hoc Bonferroni tests. Continuous data that were not normally distributed were analysed by either the Mann-Whitney-U test (2 group comparison) or the Kruskal-Wallis test (>2 groups) with post-hoc Bonferroni tests. Categorical data were expressed as percentages and compared by Chi-square and Fishers’ Exact tests. P-values were 2-sided, with odds ratios (OR) and 95% confidence intervals (CI). All these analyses were performed using the SPSS software version 22.

To assess for a differential clinical impact by various *P*. *aeruginosa* strains or subtypes, patients were categorised into one of three groups according to their *P*. *aeruginosa* typing results: (i) M3L7, (ii) non-M3L7 AUST-02 subtypes and (iii) non AUST-02 strains. Patients co-infected with both M3L7 and non M3L7 strains were classified as having M3L7. The primary outcome was death or lung transplantation from CF-related respiratory failure, while all other patients were censored (defined as alive on January 1^st^, 2011, death due to causes other than end-stage pulmonary disease or transfer from the centre). Survival time was measured in days from sample collection until endpoint events or censoring. Kaplan-Meier survival curves of unadjusted time to death or lung transplantation were generated for the three patient groups. Log-rank test was performed to assess between-group differences in survival curves. Adjustment for confounding factors was performed using univariate and multivariate Cox proportional hazards regression models. Potential confounding factors were selected *a priori* and included age, gender, CF genotype, pancreatic insufficiency, CF-related diabetes and/or liver disease, BMI, severity of airflow obstruction (categorised as mild, FEV_1_ >70%; moderate, FEV_1_ 40%-70%; severe, FEV_1_ <40%) and co-pathogens (*B*. *cepacia* complex, *S*. *aureus*, *Achromobacter* and *Aspergillus sp*.). The effect of each factor was assessed by univariate Cox-proportional analysis and factors identified as statistically significant (P-value ≤0.05) were included in the multivariate analysis. Multivariate analysis based on the Cox proportional hazard regression model was performed using a stepwise backward method based on likelihood ratios using P≤0.05 for inclusion. The proportional hazards assumption of the model was satisfied by model diagnostics including: log minus log-survival curves, diagnostic plots of Dfbeta, and estimates of martingale residues and deviance residuals. The decision to incorporate a covariate as a categorical or continuous variable was based on model diagnostic test results.

To compare the rates of disease progression between the three patient groups, multivariate repeated measures linear regression modelling was employed to account for within-patient correlation and to estimate the annual rates of change in BMI and FEV_1_ (% predicted) after adjusting for gender effect. Dunn’s test and post-hoc Bonferroni tests were then performed to compare rates of change in clinical variables between patient groups.

## Results

### *mexZ and* LasR sequence diversity; 2001–2002 and 2007–2009

Of the initial 201 *P*. *aeruginosa* isolates, 188 provided reliable sequence data for both *mexZ* and LasR regions (S1 Table) from 150 AUST-02, 17 AUST-01, 5 AUST-06, 1 AUST-11 and 15 unique strains. These 188 isolates came from 105 patients (33 children attending the RCH, 83 adults at TPCH and included 11 children who provided isolates from both centres as they transitioned from the RCH to the TPCH between the first and second survey periods; Fig 1). Overall, 25 different *mexZ* (types M1–M25; S1 Fig) and 40 LasR protein (types L1–L40; S2 Fig) sequences were identified. Interestingly, AUST-02 isolates showed only two *mexZ* types, M2 and M3 (S1 Table). Furthermore, mutations in *mexZ* (M3 type) were more likely in AUST-02 isolates in 2007–2009 (17/95, 17.9%) than 2001–2002 (3/55; 5.5%; OR = 3.8; 95%CI: 1.1–13.5) and were only identified in isolates from adults (20/115 (17.4%) submitted AUST-02 isolates) attending the TPCH, being notably absent in AUST-02 isolates collected from children at the RCH clinic.

The most common LasR type, L1, was present in 103/188 (54.8%) isolates, including 74/150 AUST-02, 13/17 AUST-01, 4/5 AUST-06, 1/1 AUST-11 and 11/15 unique isolates. The L1 sequence was homologous with that of PA01 (Genbank accession no: NC_002516.2) and considered the wild-type sequence. The remaining 39 LasR sequences (L2–L40) had substitutions and frame-shift mutations. Of these, 32 were in the 76 non-L1 AUST-02 isolates (S1 Table). Variant AUST-02 LasR sequences were more frequently observed in AUST-02 isolates collected in 2007–2009 (56/95, 58.9%) than 2001–2002 (20/55; 36.4%; OR = 2.5; 95%CI: 1.3–5.0) and were significantly more common in AUST-02 isolates from adults (67/115; 58.3%) than children (9/35; 25.7%; OR = 4.0; 95%CI: 1.7–9.4).

### Combined *mexZ* and LasR subtypes

Sixty-two *mexZ/*LasR sequence combinations (referred to as ‘subtypes’; S1 Table) were observed. Of the 150 sequenced AUST-02 isolates (79 patients), 35 different *mexZ/*LasR subtypes were identified. The M2L1 subtype was detected most commonly (72/150 isolates, 48%) and was considered the wild-type AUST-02 subtype, as it included the wild-type L1 sequence and was the most prevalent AUST-02 subtype in the early study years. Of the other 34 AUST-02 subtypes, 28 were from individuals and six were identified in clusters of two or more patients. The latter included M2L3 (four unrelated patients), M2L20 (two siblings and one unrelated patient), M2L26 (two siblings), M2L28 (two unrelated patients), M2L30 (two unrelated patients) and M3L7 (10 unrelated patients). Whilst most of these AUST-02 subtypes were confined to small patient clusters (median: 2.5 patients/cluster), 16 isolates identified as the M3L7 AUST-02 subtype were found in 10 unrelated patients whose care was based at TPCH in 2007–2009. This M3L7 AUST-02 subtype was not detected in paediatric patients attending the RCH or from the earlier collection period (2001–2002) at either centre.

### Antibiotic susceptibility

Antibiotic susceptibilities to several anti-pseudomonal antibiotics were then assessed for the 16 M3L7 isolates and in a subset of 58 isolates randomly selected from across all non-M3L7 AUST-02 subtypes (S3 Table). All 16 M3L7 isolates were MDR and demonstrated non-susceptibility (intermediate or resistant) to a significantly greater number of antibiotics than non-M3L7 AUST-02 isolates (Table 1). Except for tobramycin, M3L7 isolates also had significantly higher MIC values for all other antibiotics tested than non-M3L7 AUST-02 subtypes.

**Table 1:** Antibiotic susceptibilities of the M3L7 AUST-02 subtype compared with other non-M3L7 AUST-02 subtypes.

Antibiotic	% non-susceptible according to CLSI breakpoints^a^			Minimal Inhibitory Concentration (mg/L), Median (range)		
	M3L7 AUST-02 (n = 16) (%)	Non M3L7 AUST-02 (n = 58) (%)	RR (95% CI)	M3L7 AUST-02 (n = 16)	Non M3L7 AUST-02 (n = 58)	p-value
Meropenem	16 (100%)	31 (53.4%)	1.9 (1.5–2.4)	16 (16–32)	4 (1–10)	<0.001
Imipenem	16 (100%)	42 (72.4%)	1.4 (1.2–1.8)	32 (32–64)	8 (2–16)	<0.001
Ceftazidime	16 (100%)	39 (67.2%)	1.5 (1.2–1.8)	64 (64–128)	32 (8–64)	0.002
Ticarcillin-clavulanic acid	16 (100%)	37 (63.8%)	1.6 (1.3–1.9)	256/2 (256/2-512/2)	32/2 (4/2-64/2)	<0.001
Aztreonam	16 (100%)	23 (39.7%)	2.5 (1.8–3.5)	320 (64–512)	8 (2–40)	<0.001
Ciprofloxacin	15 (94%)	33 (56.9%)	1.6 (1.3–2.1)	4 (4–4)	2 (1–4)	0.002
Tobramycin	14 (87.5%)	39 (67.2%)	1.3 (1.0–1.7)	8 (8–16)	16 (4–512)	0.3
Colistin	12 (80%)	9 (15.5%)	4.8 (2.5–9.4)	6 (4–8)	2 (0.875–2)	<0.001
Multi-drug resistance^b^	16 (100%)	27 (46.6%)	2.1 (1.6–2.8)			

Abbreviations: CLSI, Clinical and Laboratory Standards Institute; IQR, interquartile range; RR, relative risk.

^a^ Non-susceptible (intermediate or resistant) *P*. *aeruginosa* isolates identified according to CLSI 2013 breakpoints [26] as follows: meropenem >4 mg/L; imipenem > 4mg/L; ceftazidime >16mg/L; ticarcillin-clavulanic acid >32/2 mg/L; aztreonam >16mg/L; ciprofloxacin > 2mg/L; tobramycin >8mg/L; colistin sulphate >4mg/L.

^b^ Multi-drug resistance was defined as resistance to all agents tested in two or more of the following antibiotic categories: aminoglycosides, β-lactam antibiotics and/or the fluoroquinolone, ciprofloxacin, according to criteria published by the 1994 Cystic Fibrosis Foundation Microbiology and Infectious Disease Consensus Conference [27].

### Within patient diversity and longitudinal changes in *P*. *aeruginosa* strains and subtypes between 2001–2002 and 2007–2009

In 30 patients, more than one *P*. *aeruginosa* isolate was sampled from the same sputum specimen and each of these isolates underwent *mexZ* and *lasR* sequencing. Twenty patients had isolates of the same *P*. *aeruginosa* strain with identical *mexZ*/LasR subtypes; three had different *P*. *aeruginosa* strains, and seven had two to three different AUST-02 *mexZ/*LasR subtypes within the same sputum sample.

Forty patients also had serial isolates collected at both time-points (2001–2002 and 2007–2009; Table 2A). Alterations in *P*. *aeruginosa* strains and/or intra-strain *mexZ/*LasR subtypes were seen in 17 (42.5%) of these patients (Table 2A), including two with new *P*. *aeruginosa* strains, two with changes in both *P*. *aeruginosa* strains and *mexZ/*LasR subtypes, and another 13 with changes in AUST-02 *mexZ/*LasR subtypes.

**Table 2:** Summary of longitudinal within-patient changes involving shared and unique *Pseudomonas aeruginosa* strains, including their subtypes^a^.

No. of patients	2001–2002	2007–2009	2011	Change in genotype	Type of genotypic change
**A:** ^b^					
1	M19L14 (UNIQUE)	M2L1 (AUST-02)	-^d^	Yes	Strain
1	M7L10 (UNIQUE)	M2L1 (AUST-02)	-^d^	Yes	Strain
1	M2L1 (AUST-02); M2L39 (AUST-02)	M2L1 (AUST-02); M21L2 (UNIQUE)	-^d^	Yes	Strain and Subtype
1	M2L1 (AUST-02)	M1L1 (AUST-01); M3L7 (AUST-02)	-^d^	Yes	Strain and Subtype
1	M2L1 (AUST-02)	M2L2 (AUST-02)	-^d^	Yes	Subtype
1	M2L1 (AUST-02)	M2L20 (AUST-02)	-^d^	Yes	Subtype
1	M2L1 (AUST-02)	M2L23 (AUST-02)	-^d^	Yes	Subtype
1	M2L1 (AUST-02)	M2L27 (AUST-02)	-^d^	Yes	Subtype
1	M2L1 (AUST-02)	M2L31 (AUST-02)	-^d^	Yes	Subtype
1	M2L1 (AUST-02)	M2L32 (AUST-02)	-^d^	Yes	Subtype
1	M2L1 (AUST-02)	M2L33 (AUST-02)	-^d^	Yes	Subtype
1	M2L1 (AUST-02)	M2L1 (AUST-02); M2L5 (AUST-02)	-^d^	Yes	Subtype
1	M2L22 (AUST-02)	M2L11 (AUST-02)	-^d^	Yes	Subtype
1	M2L29 (AUST-02)	M2L30 (AUST-02)	-^d^	Yes	Subtype
1	M2L3 (AUST-02)	M2L4 (AUST-02)	-^d^	Yes	Subtype
1	M3L1 (AUST-02)	M2L1 (AUST-02); M3L1 (AUST-02)	-^d^	Yes	Subtype
1	M2L1 (AUST-02); M2L25 (AUST-02)	M2L1 (AUST-02)	-^d^	Yes	Subtype
1	M8L1 (AUST-01)	M8L1 (AUST-01)	-^d^	No	No change
1	M17L1 (AUST-01)	M17L1 (AUST-01)	-^d^	No	No change
13	M2L1 (AUST-02)	M2L1 (AUST-02)	-^d^	No	No change
1	M2L3 (AUST-02)	M2L3 (AUST-02)	-^d^	No	No change
1	M2L20 (AUST-02)	M2L20 (AUST-02)	-^d^	No	No change
1	M2L24 (AUST-02)	M2L24 (AUST-02)	-^d^	No	No change
2	M2L26 (AUST-02)	M2L26 (AUST-02)	-^d^	No	No change
2	M5L1 (AUST-11)	M5L1 (AUST-11)	-^d^	No	No change
1	M12L1 (UNIQUE)	M12L1 (UNIQUE)	-^d^	No	No change
**B:** ^c^					
5	-^d^	M3L7 (AUST-02)	M3L7 (AUST-02)	No	No change
1	-^d^	AUST-06	M3L7 (AUST-02)	Yes	Strain
1	AUST-01	Non-M3L7 (AUST-02); (AUST-13)	M3L7 (AUST-02)	Yes	Strain and Subtype
1	AUST-13	AUST-01	M3L7 (AUST-02)	Yes	Strain
1	AUST-01	Non-M3L7 (AUST-02)	M3L7 (AUST-02)	Yes	Subtype
1	-^d^	UNIQUE	M3L7 (AUST-02)	Yes	Strain

^a^ AUST-01, -02, -11and -13 are strains commonly shared by Australian cystic fibrosis patients, [13] while an intra-strain ‘subtype’ is defined by *mexZ* and *LasR* sequencing.

^b^ Dataset A represents isolate strain and subtyping data derived from 40 patients at The Prince Charles Hospital (TPCH) who had serial isolates collected at two different time periods (2001–2002 and 2007–2009 respectively) available for comparison. Of the 166 patients from TPCH participating in the 2007–2009 microbiological survey targeting the ‘M3L7’ AUST-02 subtype, 126 had not provided isolates previously and are therefore not included in this table. *P*. *aeruginosa* Sequenom iPlex SNP-based genotyping results are displayed in parentheses.

^c^ Dataset B represents isolate subtyping data derived from 10/11 patients who were identified as M3L7 +ve in the subsequent 2011 microbiological survey. These 10 patients had previous *P*. *aeruginosa* strain typing data from the survey in 2007–2009, while no prior isolate strain typing data were available for the 11^th^ M3L7+ve patient who was therefore not included in the table.

^d^ No strain or subtype data available

### Point-prevalence studies of the ‘M3L7’ AUST-02 subtype in 2007–2009 and 2011

Following the initial identification of the ‘M3L7’ AUST-02 subtype, we took advantage of cross-sectional microbiological surveys [13] of all known *P*. *aeruginosa* infected CF adult patients at TPCH that were conducted during 2007–2009 and repeated in 2011. Stored isolates from these two surveys were tested by PCR assays designed specifically to detect the M3L7 subtype within the AUST-02 strains (Fig 2).

**Fig 3:**
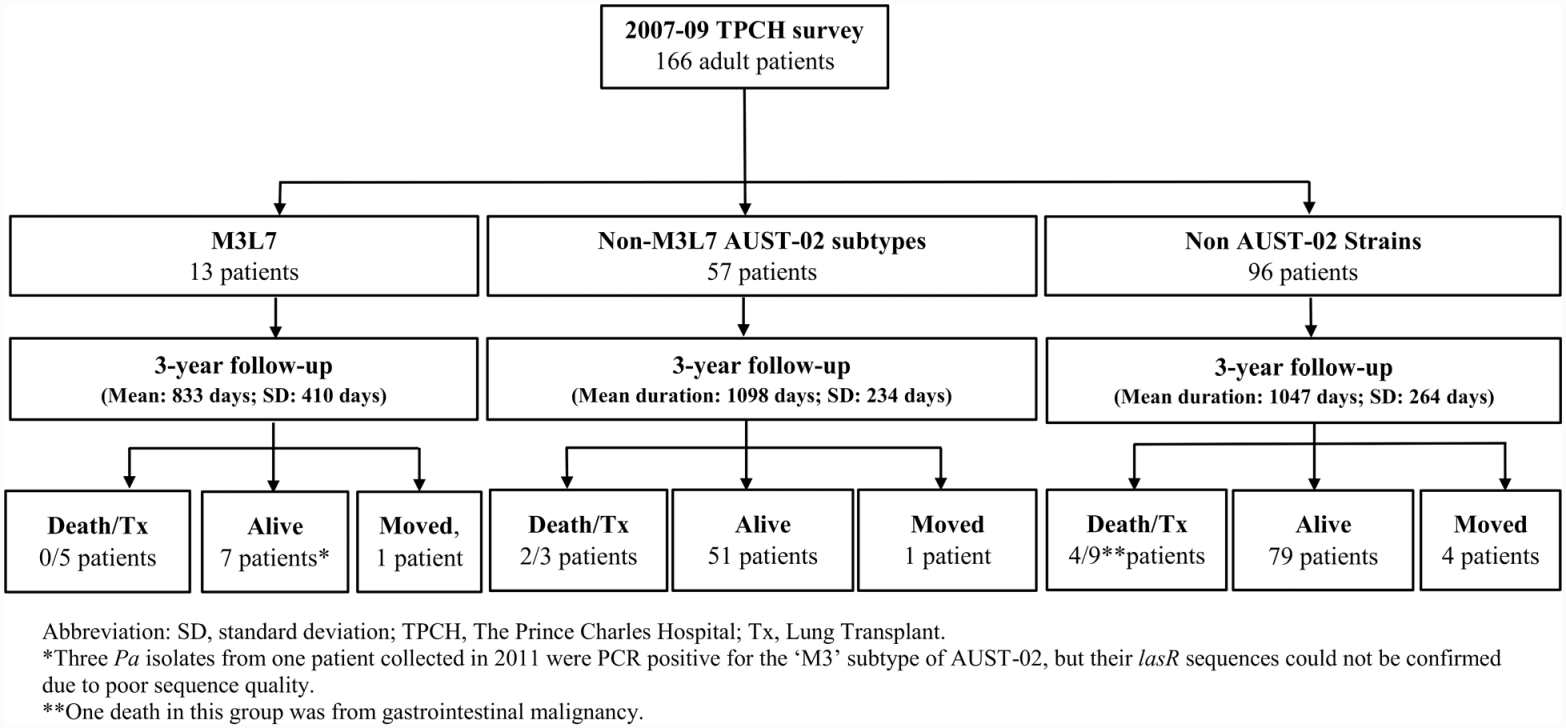
Clinical outcome of 166 TPCH adult patients within 3-years of participating in the 2007–2009 survey.

Overall, M3L7 was identified in 28/509 (5.5%) *P*. *aeruginosa* isolates from 13/170 (7.6%) patients in 2007–2009 (Fig 3). The repeat survey in 2011 identified M3L7 in 21/519 (4.0%) *P*. *aeruginosa* isolates from 11/173 (6.4%) adult patients (Table 2B). Of note, 7/13 patients with M3L7 in 2007–2009 and 7/11 patients with M3L7 in 2011 also had other co-infecting *P*. *aeruginosa* strains or subtypes. Additionally, of the 13 patients identified with M3L7 in 2007–2009, five patients underwent lung transplantation during the follow-up period and one patient moved interstate. Of the seven who had M3L7 infection in 2007 and had samples collected in 2011, five remained infected in 2011, one no longer had the M3L7 subtype detected, while another tested positive by the M3-PCR, but the LasR sequence could not be characterised because of suboptimal sequence quality. A further five patients infected previously with other *P*. *aeruginosa* strains or subtypes of AUST-02 in 2007 had acquired M3L7 by 2011 and were identified as incident cases. These five patients (three males) attended the centre between 2007–2011. They had previously harboured other *P*. *aeruginosa* strains (Table 2B) in 2007–2009, and for each patient this included either AUST-06; AUST-01; a unique strain; a non-M3L7 AUST-02 subtype, or a co-infection with non-M3L7 AUST-02 and AUST-13 strains respectively. Finally, one patient with M3L7 in 2011 had no previous strain-typing data available.

### Baseline Clinical Characteristics

Of the 170 patients surveyed in 2007–2009, 166 were available for follow-up and four were excluded as the strain types of their baseline isolates were unavailable (Fig 3). Table 3 summarises their baseline characteristics with patients categorised according to whether they had: (i) M3L7, (ii) non-M3L7 AUST-02 subtypes or (iii) non AUST-02 *P*. *aeruginosa* strains. Whilst the mean baseline FEV_1_ (% predicted) was lower in the M3L7 group (45.5%) compared with both the non M3L7 AUST-02 group (54.7%) and the non AUST-02 groups (52.8%), the differences did not reach statistical significance. However, patients with M3L7 had lower BMI and in the previous 12-months had undertaken more frequent outpatient visits than patients with non AUST-02 strains. Patients with M3L7 had also received more hospital-based IV antibiotic treatments than patients with other non-M3L7 AUST-02 subtypes and non AUST-02 strains.

**Table 3:** Baseline clinical characteristics of the 166 patients with cystic fibrosis and *Pseudomonas aeruginosa* infection from The Prince Charles Hospital surveyed in 2007–2009. ^a^

	M3L7 (n = 13)	Non-M3L7 AUST-02 subtypes (n = 57)	Non AUST-02 Strains ^b^ (n = 96)	M3L7 vs. Non-M3L7 AUST-02 subtypes	M3L7 vs. Non AUST-02 Strains
				p-value	
Age, mean (SD), years	27.3 (5.7)	26.8 (5.7)	29.6 (8.8)	0.43	0.47
Female gender, No. (%)	7.0 (53.8%)	22.0 (39.6%)	39 (40.6%)	0.36	0.39
**Genotype, no. (%)**					
Phe508del/Phe508del	6 (46.2%)	34 (59.6%)	53 (55.2%)	NS	NS
Phe508del/other	5 (38.5%)	20 (35.1%)	30 (31.3%)	NS	NS
Other/Other	-	3 (5.3%)	5 (5.2%)	NS	NS
Unknown	2 (15.4%)	0	8 (8.3%)	**0.02**	NS
**Co-morbidities, no. (%)**					
Pancreatic insufficiency	13 (100%)	56 (98.2%)	93 (96.9%)	0.63	1
Diabetes	4 (30.8%)	11 (19.3%)	22 (22.9%)	0.46	0.51
Liver disease	1 (7.7%)	8 (14.0%)	2 (2.1%)	1	0.32
BMI, mean (SD), kg/m^2^	20.2 (2.9)	21.4 (3.8)	22.1 (3.6)	0.14	**0.04**
FEV_1_% pred, mean (SD)	45.5% (23.0%)	54.7% (20.7%)	52.8 (20.1%)	0.14	0.2
**Co-infections, no. (%)**					
*Staphylococcus aureus*	1 (7.7%)	19 (33.3%)	25 (26.0%)	0.09	0.18
*Burkholderia cepacia* complex	-	1 (1.8%)	7 (7.3%)	1	0.6
*Aspergillus spp*.	-	9 (15.8%)	12 (12.5%)	0.19	0.36
*Achromobacter spp*.	2 (15.4%)	3 (5.3%)	3 (3.1%)	0.23	0.11
**Medications, no. (%)**					
Azithromycin ^c^	9 (69.0%)	22 (38.6%)	47 (49.5%)^c^	0.06	0.38
Inhaled tobramycin	3 (21.4%)	10 (18.2%)	18 (18.6%)	0.70	0.71
Inhaled colistin	1 (7.7%)	9 (15.8%)	2 (2.1%)	0.68	0.32
Inhaled dornase-alpha ^c^	8 (61.5%)	22 (38.6%)	29 (30.5%)^c^	0.21	0.08
Inhaled Hypertonic Saline	3 (23.1%)	16 (28.1%)	28 (29.2%)	1	0.76
**Centre attendance**					
No. of OPC visits past 12-months, mean (SD)	10.7 (6.3)	8.6 (4.9)	6.8 (4.7)	0.26	**0.02**
No. of IV antibiotic courses past 12-months, mean (SD)	2.6 (1.3)	1.6 (1.5)	1.7 (2.0)	**0.01**	**0.01**
Days on IV antibiotics past 12-months, mean (SD)	40.2 (26.3)	24.3 (27.7)	24.1 (33.6)	**0.01**	**0.005**
No. of patients lost to follow-up	1	-	2	-	-

^a^ Abbreviations: BMI, body mass index; FEV_1_, forced expiratory volume in 1 second; % pred, percent predicted; IV, intravenous; SD, standard deviation.

^b^ The 96 patients had unique (43), AUST-01 (18); AUST-06 (10); AUST-07 (5); AUST-11 (3); Clone C (3); AUST-13 (2), AUST-04 (2), AUST-20 (2), AUST-22 (2), AUST-24 (2), AUST-05 (1), AUST-14 (1), AUST-21 (1) and Liverpool epidemic (1) strains.

^c^ Data from one patient was unavailable for azithromycin and inhaled dornase-alpha treatment.

### Clinical Outcomes

**Fig 4:**
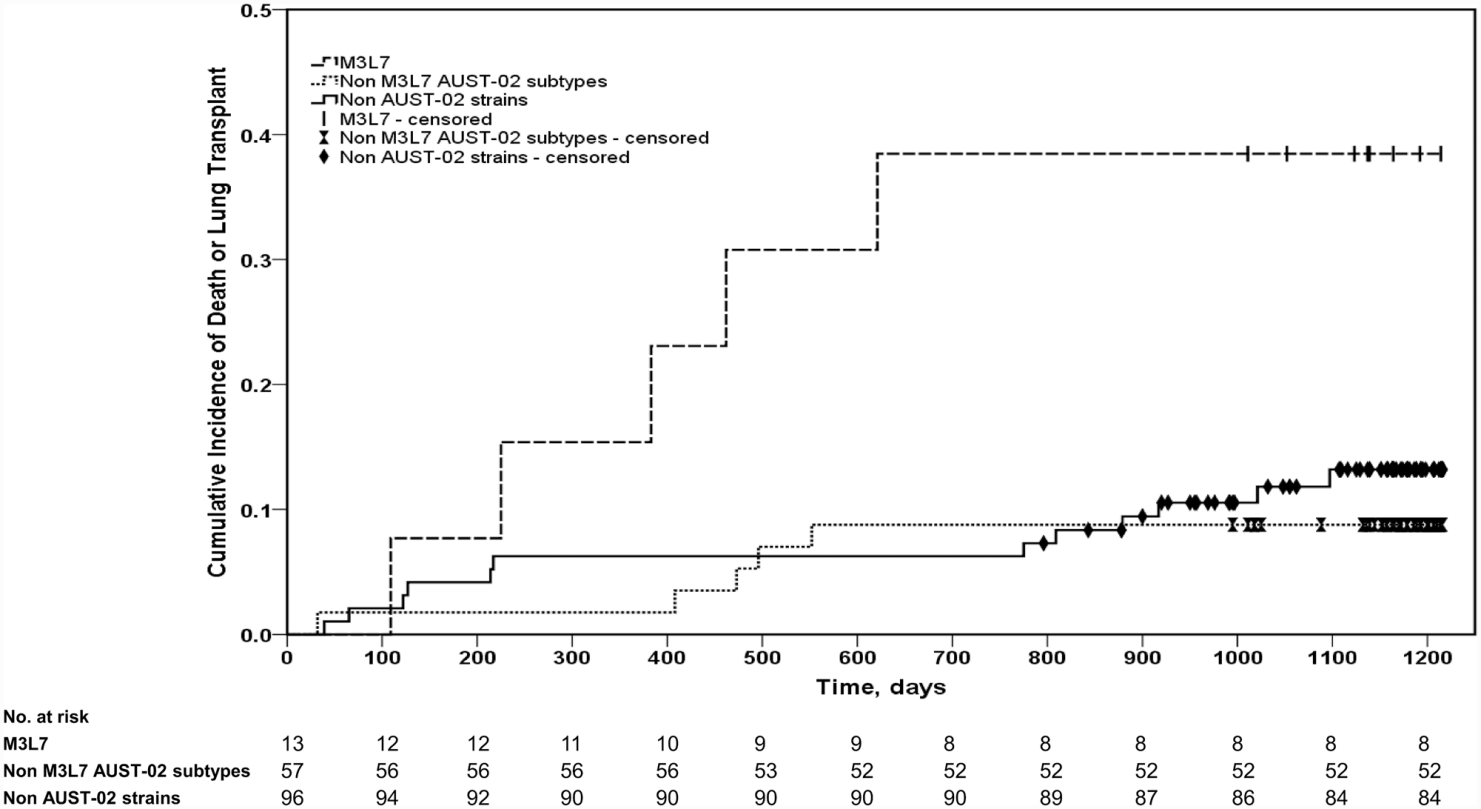
Kaplan-Meier survival analysis comparing unadjusted time to death or lung transplantation for 13 patients with M3L7, 57 with non-M3L7 AUST-02 and 96 patients with non-AUST-02 *Pseudomonas aeruginosa* strains.

The mean (±SD) follow-up period for the entire cohort was 1067 (±242) days. During this time, 6/166 (3.6%) patients died and 17/166 (10.2%) underwent lung transplantation (Fig 3). This included 5/13 (38.5%) with M3L7 (5 transplanted), 5/57 (9%) with non-M3L7 AUST-02 subtypes (2 died, 3 transplanted), and 13/96 (13.5%) with non-AUST-02 strains (4 died, 9 transplanted). Infection with M3L7 was associated with a significantly greater 3-year risk of death/lung transplantation compared with patients infected with either non-M3L7 AUST-02 subtypes (unadjusted hazards ratio (HR) = 5.4; 95%CI: 1.56–18.67; adjusted HR = 9.4; 95%CI: 2.2–39.2) or non-AUST-02 strains (unadjusted HR = 3.7; 95%CI: 1.30–10.4; adjusted HR = 4.8; 95%CI: 1.4–16.2) adjusted for the effects of age, BMI and severity of airway disease (Fig 4). In contrast, no differences were observed in the 3-year adjusted HR of death/lung transplantation between patients infected with non-M3L7 AUST-02 subtypes and non-AUST-02 strains.

While none died within the M3L7 group, two died from respiratory failure in the non-M3L7 AUST-02 subtype group (one was ineligible for transplantation because of *B*. *cepacia* complex, the other declined a transplant) and another four (4%) died in the non AUST-02 group. Three of the four deaths in the non AUST-02 group were attributable to severe CF-related airways disease, including two who died on the transplant waiting list and another who was ineligible for transplantation due to co-infection with *B*. *cepacia* complex. The other death in the non AUST-02 group was from metastatic gastrointestinal malignancy.

The secondary outcomes during the 3-year follow-up for the three patient groups are presented in Table 4. Adults harbouring M3L7 in 2007–2009 had greater rates of decline in BMI and lung function, although these did not reach statistical significance. It would also appear from the univariate analysis that those infected with M3L7 had a significantly greater number of hospitalisation days for IV antibiotics annually than either of the other two patient groups (Table 4).

**Table 4:** Disease progression and treatment burden (2007–2009 and 2011) by *Pseudomonas aeruginosa* strain and subtype groupings.

	*P*. *aeruginosa* strain (n = 166)			Median Difference (IQR)					
	M3L7 (n = 13)	Non-M3L7 AUST-02 subtypes ^a^ (n = 57)	Non-AUST-02 Strains (n = 96)	M3L7 vs. Non-M3L7 AUST-02 subtypes	*p-value*	M3L7 vs. Non-AUST-02 Strains	*p-value*	Non-M3L7 AUST-02 subtypes vs. Non-AUST-02 strains	*p-value*
Median annual rate of change in BMI (kg/m^2^) (IQR)	-0.03 (-0.63–0.15)	0.05 (-0.09–0.29)	0.15 (-0.08–0.37)	-0.08 (-0.23–0.01)	0.5	-0.18 (-0.33 –-0.10)	0.3	-0.10 (-0.13 –-0.07)	0.07
Median annual rate of change in FEV_1_ (% pred) (IQR)	-2.2 (-3.1 –-1.6)	-1.4 (-2,2–0.2)	-1.2 (-2.7–0.1)	-0.8 (-1.3 − -0.4)	0.2	-1.0 (-1.3 –-0.5)	0.1	-0.2 (-0.3–0.2)	1.0
Median annual hospital-based IV antibiotic days (IQR)	27.5 (20.0–9.8)	9.3 (5.1–21.5)	10.0 (3.6–25.3)	18.3 (4.3–32.2)	**0.02**	15.0 (3.0–24.0)	**0.04**	0.0 (-3.5–3.8)	1.0
Median annual outpatient reviews (IQR)	6.0 (3.3–8.8)	7.3 (3.8–10.0)	5.3 (2.8–8.8)	-1.3 (-5.0–2.7)	0.9	0.5 (-2.0–3.3)	0.6	1.0 (-0.4–2.5)	0.3

Abbreviations: BMI, body mass index; FEV_1_, forced expiratory volume in 1 second; % pred, percent predicted; IQR, interquartile range; IV, intravenous.

* Dunn’s test and post-hoc Bonferroni test was used for comparison of mean annual rate of change in FEV_1_(% predicted) and BMI. Kruskal-Wallis with post-hoc Bonferroni test was used for comparison of mean annual hospital-based IV antibiotics days and mean annual outpatient clinic reviews.

^**a**^ Information on outpatient clinic visits and hospitalisation history was unavailable for two patients in this group.

## Discussion

Microevolution of the *P*. *aeruginosa* genome is an important pathoadaptive process within the CF airways, characterised by extensive genotypic and phenotypic diversification [16, 17, 19, 20]. However, the potential clinical implications of such ongoing microevolution within common CF-specific *P*. *aeruginosa* shared strains in terms of cross-transmission and impact on clinical outcomes remains unclear. Consistent with earlier studies, we observed *mexZ* and LasR sequence diversity among the entire 188 isolate collection [16, 21, 22, 29, 30]. In particular, we observed the increased frequency of AUST-02 LasR sequence variants in adult compared with paediatric patients. Likewise, longitudinal changes in LasR sequence were also observed in isolates from approximately 40% of study patients. This observation is supported indirectly by other studies linking *lasR* loss-of-function mutations with the transition from early to chronic *P*. *aeruginosa* infection and accelerated pulmonary function decline in CF [22, 25, 30]. The *lasR* gene is a key transcriptional regulator of quorum sensing in *P*. *aeruginosa* and its mutational inactivation is associated with attenuated virulence, host immune evasion and a growth advantage from utilising the specific carbon and nitrogen sources available in the amino-acid rich CF airway secretions [31]. Inactivation of the *lasR* gene is also associated with increased β-lactamase activity and ceftazidime tolerance [31], which facilitates its adaptation to the CF airway.

During the 10-year study period we observed the emergence and expansion of an intra-strain AUST-02 subtype (M3L7), characterised by an enhanced MDR phenotype, which superseded its ancestral wild-type AUST-02 strain and other AUST-02 subtypes. M3L7 appears to have retained its transmissibility, which presumably contributed to the additional incident cases observed within the adult CF clinic between 2007 and 2011. Our results also demonstrated significant differences in clinical trajectories between patients infected with different AUST-02 intra-strain subtypes.

As respiratory failure is the major cause of death in patients with CF, and lung transplantation is a viable treatment for end-stage CF lung disease, like others we chose time to either death or lung transplantation as the primary endpoint events in our survival analysis [11]. Infection with the M3L7 AUST-02 subtype was associated with a nine-fold increase in the overall 3-year risk of death or lung transplantation compared with other non-M3L7 AUST-02 subtypes and a five-fold increase in risk when compared with non AUST-02 strains. In contrast, no significant differences were detected between patients infected with non M3L7 AUST-02 subtypes and non AUST-02 strains. M3L7 infected patients also had significantly greater requirement for hospitalisation for IV antibiotics at baseline and follow-up.

Therefore, our results suggested there might be differences in the clinical outcomes according to intra-strain subtypes within this commonly shared *P*. *aeruginosa* strain. The mechanisms underlying the association of the M3L7 subtype with poorer clinical outcomes remain uncertain. Possible explanations include its MDR phenotype could lead to less effective antibiotic treatment or it could simply represent a transmissible strain found more frequently in those with severe CF pulmonary disease following their increased healthcare requirements and exposure to other infected patients.

The latter is suggested by the lower baseline FEV_1_ (% predicted) in the M3L7 infected patients compared with the other groups of *P*. *aeruginosa* infected patients, although with the small numbers involved this did not reach statistical significance. Whilst there were no deaths amongst M3L7 infected patients, a significantly greater proportion of M3L7 infected patients required lung transplantation than observed with the other groups. It is possible therefore that either the increased IV antibiotics requirement associated with M3L7 infection at both baseline and follow-up reflected the clinical impact of the MDR M3L7 subtype or, alternatively, the intensified IV antibiotic usage in the pre-transplant period or a combination of both. Furthermore, greater antibiotic exposure in patients with M3L7 infection may have also contributed to the development of the MDR phenotype observed with this subtype. Whole genome sequencing studies are planned to explore these possibilities.

Nevertheless, until the above questions are resolved, the emergence and ongoing transmission of this AUST-02 subtype raises important questions over current infection control practices. There is now evidence that *P*. *aeruginosa* survives in cough aerosols up to 4-metres and for as long as 45-minutes, suggesting cough bioaerosols as a possible transmission pathway [32–34]. Strain-specific cohort segregation practiced by some Australian and international CF centres has interrupted acquisition of their targeted strains [35–37]. However, as reported previously with *B*. *cenocepacia* [38], cohort-segregation without additional strain-typing risks acquiring more virulent (epidemic) subtypes. Our results agree with recent reports of cross-infection with intra-strain sublineages of the highly successful LES strain in CF patients cohorted according to strain-based segregation policies at an English CF centre [39]. We provide further observations of possible adverse clinical outcomes associated with the spread of an emerging MDR intra-strain subtype of AUST-02 at an Australian CF centre. These findings support the United Kingdom CF Standards of Care [40] and CF Foundation Infection Control Guidelines [41] that recommend all hospitalised patients remain in single rooms to minimise cross-infection. Australian CF centres are now optimising hospital-based and outpatient care to limit contact between patients.

This study does however have some important limitations. First, the *P*. *aeruginosa* isolates originated from three different projects. Whilst none of these studies were designed initially to monitor microevolution of bacterial strains, combining these historical sample banks has provided us with an ideal opportunity to examine the pattern of intra-strain diversification within the AUST-02 strain using a large number of isolates collected from a representative population of Queensland paediatric and adult CF patients infected over a decade. Second, as a maximum of three colonies were selected from sputum culture plates, the numbers of strains and their subtypes within individuals may have been substantially underestimated. However, we believe the limitation in the sampling depth is partially compensated by the extent and period of sampling, which has enabled us to assess the between-patient, rather than within-patient, AUST-02 population heterogeneity. Furthermore, a small number of patients appeared to have undergone repeated new strain acquisition at the three different time-points (2001–2002; 2007–2009; 2011) (Table 3). Given most patients retained the same strains for prolonged periods, this observation may relate to either repeated strain replacements from multiple cross-infection events or under-sampling of co-infecting strains. Thirdly, the relatively small number of patients infected with the M3L7 subtype is reflected in the broad 95%CIs around risk estimates, which means results must be interpreted cautiously. In addition, due to the paucity of *P*. *aeruginosa* strain data prior to the ACPinCF study (2007–2009), the date of individual M3L7 acquisition remains unknown. Thus our outcome analysis was limited to prevalent rather than incident cases. Further prospective longitudinal studies are now needed to help clarify whether acquiring the M3L7 strain is associated with adverse clinical outcomes. Finally, we used *mexZ* and *lasR* sequencing as a fine typing tool to detect intra-strain diversity. While these two genes are frequent mutation targets contributing to early adaptive evolution [16, 21, 22, 29] we observed limited sequence diversity in the *mexZ* gene in AUST-02 strains. Such restricted genomic diversity agrees with reports of another persistent, shared CF strain (DK2) from Denmark, which suggest that mutations in *P*. *aeruginosa* strains highly adapted to the CF lung are constrained [22, 42]. Further study of these successful CF strains requires whole genome sequencing techniques that provide greater resolution than simply targeting selected genes.

In summary, our study showed the emergence and suspected person-to-person transmission of a MDR intra-strain subtype (M3L7) within AUST-02, the dominant *P*. *aeruginosa* strain in our adult CF clinic. This subtype was associated with poorer clinical outcomes than its ancestral strain. These data also suggest that commonly shared *P*. *aeruginosa* strains can evolve into more resistant subtypes, while also retaining the ability to infect other CF patients. This has important infection control implications when adopting *P*. *aeruginosa* strain-specific segregation strategies in the CF clinic. Based on the results from our current study and other studies from our group [13], a new purpose-designed CF ward with single room facilities for all CF patients hospitalised for IV antibiotics was commissioned in 2014, allowing total segregation of all patients from one another during hospitalisation. Studies are planned to characterise the underlying mechanisms associated with resistance, virulence and the metabolic profile of this newly described AUST-02 subtype.

## Supporting Information

S1 Fig Nucleotide sequence variation among the 25 *Pseudomonas aeruginosa mexZ* gene sequences. (PDF)

S2 Fig Sequence variation among the 40 *Pseudomonas aeruginosa* LasR amino acid sequences. (PDF)

S1 Table Intra-strain diversity of the *mexZ* and LasR sequences among the 188 *Pseudomonas aeruginosa* isolates from 105 patients; 2001–2002 and 2007–2009. (DOCX)

S2 Table Primer sets used in *mexZ* and *lasR* sequencing. (DOCX)

S3 Table Minimum inhibitory concentrations (mg/L), number of non-susceptible antibiotic results and multidrug resistance among the 74 AUST-02 strain isolates. (DOCX)

## Abbreviations

BMI: body-mass-index
CLSI: Clinical and Laboratory Standards Institute
CI: confidence interval
CF: cystic fibrosis
ERIC-PCR: enterobacterial repetitive intergenic consensus-polymerase chain reaction
FEV_1_: forced expiratory volume in the first second
HR: hazards ratio
IV: intravenous
IQR: interquartile range
LES: Liverpool epidemic strain
MIC: minimum inhibitory concentration
MDR: multi-drug resistant
OR: odds ratio
PCR: polymerase chain reaction
RCH: Royal Children’s Hospital
RR: relative risk
SNP: single nucleotide polymorphism
SD: standard deviation
TPCH: The Prince Charles Hospital

## References

[1] Burns JL, Gibson RL, McNamara S, Yim D, Emerson J, Rosenfeld M, et al Longitudinal assessment of *Pseudomonas aeruginosa* in young children with cystic fibrosis. J Infect Dis. 2001;183(3):444–52. 11133376 10.1086/318075

[2] Kidd TJ, Ramsay KA, Vidmar S, Carlin JB, Bell SC, Wainwright CE, et al *Pseudomonas aeruginosa* genotypes acquired by children with cystic fibrosis by age 5-years. J Cyst Fibros. 2015;14(3):361–9. 10.1016/j.jcf.2014.12.007 25563522

[3] Mogayzel PJ Jr., Naureckas ET, Robinson KA, Brady C, Guill M, Lahiri T, et al Cystic Fibrosis Foundation pulmonary guideline. pharmacologic approaches to prevention and eradication of initial Pseudomonas aeruginosa infection. Ann Am Thorac Soc. 2014;11(10):1640–50. 10.1513/AnnalsATS.201404-166OC 25549030

[4] Hauser AR, Jain M, Bar-Meir M, McColley SA. Clinical significance of microbial infection and adaptation in cystic fibrosis. Clin Microbiol Rev. 2011;24(1):29–70. 10.1128/CMR.00036-10 21233507 PMC3021203

[5] Kerem E, Viviani L, Zolin A, MacNeill S, Hatziagorou E, Ellemunter H, et al Factors associated with FEV_1_ decline in cystic fibrosis: analysis of the ECFS patient registry. Eur Respir J. 2014;43(1):125–33. 10.1183/09031936.00166412 23598952

[6] Kidd TJ, Ritchie SR, Ramsay KA, Grimwood K, Bell SC, Rainey PB. *Pseudomonas aeruginosa* exhibits frequent recombination, but only a limited association between genotype and ecological Setting. PLoS One. 2012;7(9):e44199 10.1371/journal.pone.0044199 22970178 PMC3435406

[7] Fothergill JL, Walshaw MJ, Winstanley C. Transmissible strains of *Pseudomonas aeruginosa* in cystic fibrosis lung infections. Eur Respir J. 2012;40(1):227–38. 10.1183/09031936.00204411 22323572

[8] Scott FW, Pitt TL. Identification and characterization of transmissible *Pseudomonas aeruginosa* strains in cystic fibrosis patients in England and Wales. J Med Microbiol. 2004;53(Pt 7):609–15. 15184530 10.1099/jmm.0.45620-0

[9] Ashish A, Shaw M, Winstanley C, Ledson MJ, Walshaw MJ. Increasing resistance of the Liverpool Epidemic Strain (LES) of *Pseudomonas aeruginosa (Psa)* to antibiotics in cystic fibrosis (CF)—a cause for concern? J Cyst Fibros. 2012;11(3):173–9. 10.1016/j.jcf.2011.11.004 22146482

[10] Al-Aloul M, Crawley J, Winstanley C, Hart CA, Ledson MJ, Walshaw MJ. Increased morbidity associated with chronic infection by an epidemic Pseudomonas aeruginosa strain in CF patients. Thorax. 2004;59(4):334–6. 15047956 10.1136/thx.2003.014258 PMC1763796

[11] Aaron SD, Vandemheen KL, Ramotar K, Giesbrecht-Lewis T, Tullis E, Freitag A, et al Infection with transmissible strains of *Pseudomonas aeruginosa* and clinical outcomes in adults with cystic fibrosis. JAMA. 2010;304(19):2145–53. 10.1001/jama.2010.1665 21081727

[12] Jones AM, Dodd ME, Morris J, Doherty C, Govan JR, Webb AK. Clinical outcome for cystic fibrosis patients infected with transmissible Pseudomonas aeruginosa: an 8-year prospective study. Chest. 2010;137(6):1405–9. 10.1378/chest.09-2406 20081099

[13] Kidd TJ, Ramsay KA, Hu H, Marks GB, Wainwright CE, Bye PT, et al Shared *Pseudomonas aeruginosa* genotypes are common in Australian cystic fibrosis centres. Eur Respir J. 2013;41(5):1091–100. 10.1183/09031936.00060512 22878877

[14] Kidd TJ, Magalhaes RJ, Paynter S, Bell SC, Group ACI. The social network of cystic fibrosis centre care and shared *Pseudomonas aeruginosa* strain infection: a cross-sectional analysis. Lancet Respir Med. 2015;3(8):640–50. 10.1016/S2213-2600(15)00228-3 26208994

[15] O'Carroll MR, Syrmis MW, Wainwright CE, Greer RM, Mitchell P, Coulter C, et al Clonal strains of *Pseudomonas aeruginosa* in paediatric and adult cystic fibrosis units. Eur Respir J. 2004;24(1):101–6. 15293611 10.1183/09031936.04.00122903

[16] Smith EE, Buckley DG, Wu Z, Saenphimmachak C, Hoffman LR, D'Argenio DA, et al Genetic adaptation by *Pseudomonas aeruginosa* to the airways of cystic fibrosis patients. Proc Natl Acad Sci U S A. 2006;103(22):8487–92. 16687478 10.1073/pnas.0602138103 PMC1482519

[17] Chung JC, Becq J, Fraser L, Schulz-Trieglaff O, Bond NJ, Foweraker J, et al Genomic variation among contemporary *Pseudomonas aeruginosa* isolates from chronically infected cystic fibrosis patients. J Bacteriol. 2012;194(18):4857–66. 10.1128/JB.01050-12 22753054 PMC3430303

[18] Workentine ML, Sibley CD, Glezerson B, Purighalla S, Norgaard-Gron JC, Parkins MD, et al Phenotypic heterogeneity of *Pseudomonas aeruginosa* populations in a cystic fibrosis patient. PLoS One. 2013;8(4):e60225 10.1371/journal.pone.0060225 23573242 PMC3616088

[19] Foweraker JE, Laughton CR, Brown DF, Bilton D. Phenotypic variability of *Pseudomonas aeruginosa* in sputa from patients with acute infective exacerbation of cystic fibrosis and its impact on the validity of antimicrobial susceptibility testing. J Antimicrob Chemother. 2005;55(6):921–7. 15883175 10.1093/jac/dki146

[20] Mowat E, Paterson S, Fothergill JL, Wright EA, Ledson MJ, Walshaw MJ, et al *Pseudomonas aeruginosa* population diversity and turnover in cystic fibrosis chronic infections. Am J Respir Crit Care Med. 2011;183(12):1674–9. 10.1164/rccm.201009-1430OC 21297072

[21] Jeukens J, Boyle B, Bianconi I, Kukavica-Ibrulj I, Tummler B, Bragonzi A, et al Complete Genome Sequence of Persistent Cystic Fibrosis Isolate Pseudomonas aeruginosa Strain RP73. Genome Announc. 2013;1(4). 10.1128/genomeA.00568-13 PMC3731849 23908295

[22] Marvig RL, Sommer LM, Molin S, Johansen HK. Convergent evolution and adaptation of *Pseudomonas aeruginosa* within patients with cystic fibrosis. Nat Genet. 2015;47(1):57–64. 10.1038/ng.3148 25401299

[23] Fernandez L, Hancock RE. Adaptive and mutational resistance: role of porins and efflux pumps in drug resistance. Clin Microbiol Rev. 2012;25(4):661–81. 10.1128/CMR.00043-12 23034325 PMC3485749

[24] Folkesson A, Jelsbak L, Yang L, Johansen HK, Ciofu O, Hoiby N, et al Adaptation of *Pseudomonas aeruginosa* to the cystic fibrosis airway: an evolutionary perspective. Nat Rev Microbiol. 2012;10(12):841–51. 10.1038/nrmicro2907 23147702

[25] Hoffman LR, Kulasekara HD, Emerson J, Houston LS, Burns JL, Ramsey BW, et al *Pseudomonas aeruginosa lasR* mutants are associated with cystic fibrosis lung disease progression. J Cyst Fibros. 2009;8(1):66–70. 10.1016/j.jcf.2008.09.006 18974024 PMC2631641

[26] Syrmis MW, Kidd TJ, Moser RJ, Ramsay KA, Gibson KM, Anuj S, et al A comparison of two informative SNP-based strategies for typing *Pseudomonas aeruginosa* isolates from patients with cystic fibrosis. BMC Infect Dis. 2014;14(1):307. 24902856 10.1186/1471-2334-14-307 PMC4053291

[27] CLSI. Clinical and Laboratory Standards Institute. Performance Standards for Antimicrobial Susceptibility Testing; Twenty-Third Informational Supplement CLSI document M100-S23. Wayne, Pennsylvania: Clinical and Laboratory Standards Institute; 2013. 2013.

[28] Foundation CF. MIcrobiology and infections disease in cystic fibrosis. Microbiology and Infectious Disease Consensus Conference; Bethesda, MD1994.

[29] Qin X, Zerr DM, McNutt MA, Berry JE, Burns JL, Kapur RP. *Pseudomonas aeruginosa* syntrophy in chronically colonized airways of cystic fibrosis patients. Antimicrob Agents Chemother. 2012;56(11):5971–81. 10.1128/AAC.01371-12 22964251 PMC3486535

[30] Hogardt M, Heesemann J. Adaptation of *Pseudomonas aeruginosa* during persistence in the cystic fibrosis lung. Int J Med Microbiol. 2010;300(8):557–62. 10.1016/j.ijmm.2010.08.008 20943439

[31] D'Argenio DA, Wu M, Hoffman LR, Kulasekara HD, Deziel E, Smith EE, et al Growth phenotypes of *Pseudomonas aeruginosa lasR* mutants adapted to the airways of cystic fibrosis patients. Mol Microbiol. 2007;64(2):512–33. 17493132 10.1111/j.1365-2958.2007.05678.x PMC2742308

[32] Knibbs LD, Johnson GR, Kidd TJ, Cheney J, Grimwood K, Kattenbelt JA, et al *Viability of Pseudomonas aeruginosa* in cough aerosols generated by persons with cystic fibrosis. Thorax. 2014;69(8):740–5. 10.1136/thoraxjnl-2014-205213 24743559 PMC4112489

[33] Wainwright CE, France MW, O'Rourke P, Anuj S, Kidd TJ, Nissen MD, et al Cough-generated aerosols of *Pseudomonas aeruginosa* and other Gram-negative bacteria from patients with cystic fibrosis. Thorax. 2009;64(11):926–31. 10.1136/thx.2008.112466 19574243 PMC2764123

[34] Jones AM, Govan JR, Doherty CJ, Dodd ME, Isalska BJ, Stanbridge TN, et al Identification of airborne dissemination of epidemic multiresistant strains of *Pseudomonas aeruginosa* at a CF centre during a cross infection outbreak. Thorax. 2003;58(6):525–7. 12775867 10.1136/thorax.58.6.525 PMC1746694

[35] Griffiths AL, Jamsen K, Carlin JB, Grimwood K, Carzino R, Robinson PJ, et al Effects of segregation on an epidemic *Pseudomonas aeruginosa* strain in a cystic fibrosis clinic. Am J Respir Crit Care Med. 2005;171(9):1020–5. 15709051 10.1164/rccm.200409-1194OC

[36] Ashish A, Shaw M, Winstanley C, Humphreys L, Walshaw MJ. Halting the spread of epidemic Pseudomonas aeruginosa in an adult cystic fibrosis centre: a prospective cohort study. JRSM Short Rep. 2013;4(1):1 10.1258/shorts.2012.012018 23413403 PMC3572656

[37] Luna RA, Millecker LA, Webb CR, Mason SK, Whaley EM, Starke JR, et al Molecular epidemiological surveillance of multidrug-resistant *Pseudomonas aeruginosa* isolates in a pediatric population of patients with cystic fibrosis and determination of risk factors for infection with the Houston-1 strain. J Clin Microbiol. 2013;51(4):1237–40. 10.1128/JCM.02157-12 23303498 PMC3666811

[38] Ledson MJ, Gallagher MJ, Corkill JE, Hart CA, Walshaw MJ. Cross infection between cystic fibrosis patients colonised with *Burkholderia cepacia* . Thorax. 1998;53(5):432–6. 9708241 10.1136/thx.53.5.432 PMC1745231

[39] Williams D, Evans B, Haldenby S, Walshaw MJ, Brockhurst MA, Winstanley C, et al Divergent, coexisting *Pseudomonas aeruginosa* lineages in chronic cystic fibrosis lung infections. Am J Respir Crit Care Med. 2015;191(7):775–85. 10.1164/rccm.201409-1646OC 25590983 PMC4407486

[40] Trust CF. Standards for the Clinical Care of Children and Adults with Cystic Fibrosis in the UK. Second Edition. December 2011.

[41] Saiman L, Siegel JD, LiPuma JJ, Brown RF, Bryson EA, Chambers MJ, et al Infection prevention and control guideline for cystic fibrosis: 2013 update. Infect Control Hosp Epidemiol. 2014;35 Suppl 1:S1–S67. 10.1086/676882 25025126

[42] Yang L, Jelsbak L, Marvig RL, Damkiaer S, Workman CT, Rau MH, et al Evolutionary dynamics of bacteria in a human host environment. Proc Natl Acad Sci U S A. 2011;108(18):7481–6. 10.1073/pnas.1018249108 21518885 PMC3088582

