# Supplementary material for: Genotypic Diversity within a Single Pseudomonas aeruginosa Strain Commonly Shared by Australian Patients with Cystic Fibrosis
Source: PLoS One. 2015 Dec 3;10(12):e0144022. doi: 10.1371/journal.pone.0144022 (PMC4669131; doi:10.1371/journal.pone.0144022)
Supplement: S1 Fig — (PDF) [file pone.0144022.s001.pdf]

```
                 10        20        30        40        50        60        70        80        90       100
        ....|....|....|....|....|....|....|....|....|....|....|....|....|....|....|....|....|....|....|....|
PA01    TATAATCAATCATGAGTGATTAATTAATTGGCGTTTCTGTAACATATCCTTATGATCTGCGGCGCCTTTCCC-TTGTGAGGACGTTCAGTGGCCAGGAAA
MEX001  ........................................................................-...........................
MEX002  ........................................................................-...........................
MEX003  ........................................................................-...........................
MEX004  ........................................................................-...........................
MEX005  ........................................................................-...........................
MEX006  ........................................................................-...........................
MEX007  .................................................................T......-...........................
MEX008  ........................................................................T...........................
MEX009  ...............A........................................................-...........................
MEX010  ........................................................................-...........................
MEX011  ........................................................................T...........................
MEX012  ........................................................................-...........................
MEX013  ........................................................................T...........................
MEX014  ........................................................................-...........................
MEX015  ........................................................................-...........................
MEX016  ........................................................................C...........................
MEX017  ........................................................................T...........................
MEX018  ........................................................................-...........................
MEX019  ........................................................................-...........................
MEX020  ........................................................................-...........................
MEX021  ........................................................................-...........................
MEX022  ........................................................................-...........................
MEX023  ........................................................................T...........................
MEX024  ........................................................................T...........................
MEX025  ........................................................................T...........................

                110       120       130       140       150       160       170       180       190       200
        ....|....|....|....|....|....|....|....|....|....|....|....|....|....|....|....|....|....|....|....|
PA01    ACCAAAGAGGAATCCCAGAAAACCCGCGACGGCATACTCGATGCCGCCGAGCGGGTTTTCCTGGAAAAGGGCGTGGGCACCACTGCCATGGCCGACCTGG
MEX001  ....................................................................................................
MEX002  ................................................................................T..................A
MEX003  ......................A.........................................................T...................
MEX004  ....................................................................................................
MEX005  ................................................................................T...................
MEX006  ................................................................................T...................
MEX007  ................................................................................T...................
MEX008  ....................................................................................................
MEX009  ................................................................................T...................
MEX010  ................................................................................T...................
MEX011  ....................................................................................................
MEX012  ................................................................................T...................
MEX013  ....................................................................................................
MEX014  ....................................................................................................
MEX015  ................................................................................T...................
MEX016  ....................................................................................................
MEX017  ....................................................................................................
MEX018  ................................................................................T...................
MEX019  ................................................................................T...................
MEX020  ................................................................................T...................
MEX021  ....................................................................................................
MEX022  ................................................................................T...................
MEX023  ............................................................................A.......................
MEX024  ....................................................................................................
MEX025  ....................................................................................................

                210       220       230       240       250       260       270       280       290       300
        ....|....|....|....|....|....|....|....|....|....|....|....|....|....|....|....|....|....|....|....|
PA01    CGGACGCCGCCGGGGTTTCTCGCGGTGCGGTCTACGGCCACTACAAGAA------CAAGATCGAGGTCTGCCTGGCGATGTGCGACCGCGCCTTCGGCCA
MEX001  .................................................------...............T.............................
MEX002  .................................................------.............................................
MEX003  .................................................------.............................................
MEX004  .................................................------.............................................
MEX005  .................................................------.............................................
MEX006  .................................................CAAGAA.............................................
MEX007  ...........................T.....................C------............................................
MEX008  .................................................------.............................................
MEX009  ................................................T------.............................................
MEX010  .................................................------.............................................
MEX011  .................................................CAAGAA.............................................
MEX012  .......................A...................T.....------.............................................
MEX013  .................................................------.............................................
MEX014  .................................................------.............................................
MEX015  .................................................C------............................................
MEX016  .................................................------.............................................
```

```
MEX017  .................................................------.............................................
MEX018  .................................................C------............................................
MEX019  .................................................------.............................................
MEX020  .................................................------.............................................
MEX021  .................................................------........................................T....
MEX022  .................................................------.............................................
MEX023  .................................................------.............................................
MEX024  .................................................------.............................................
MEX025  .................................................------.............................................

                    310       320       330       340       350       360       370       380       390       400
        ....|....|....|....|....|....|....|....|....|....|....|....|....|....|....|....|....|....|....|....|
PA01    GATCGAGGTACCCGACGAAAACGCCAGGGTGCCGGCGCTGGATATCCTCCTGCGCGCCGGCATGGGCTTTCTCCGCCAGTGCTGCGAGCCCGGTTCGGTG
MEX001  ....................................................................................................
MEX002  ...T................................................................................................
MEX003  ...T................................................................................................
MEX004  ....................................................................................................
MEX005  ...........................................................................T........................
MEX006  ....................................................................................................
MEX007  ....................................................................................................
MEX008  ....................................................................................................
MEX009  ....................................................................................................
MEX010  ...........................................................................T........................
MEX011  ....................................................................................................
MEX012  ..........................................C.........................................................
MEX013  ....................................................................................................
MEX014  ....................................................................................................
MEX015  ....................................................................................................
MEX016  ....................................................................................................
MEX017  ....................................................................................................
MEX018  ....................................................................................................
MEX019  ....................................................................................................
MEX020  ....................................................................................................
MEX021  ....................................................................................................
MEX022  ....................................................................................................
MEX023  ....................................................................................................
MEX024  ....................................................................................................
MEX025  ....................................................................................................

                    410       420       430       440       450       460       470       480       490       500
        ....|....|....|....|....|....|....|....|....|....|....|....|....|....|....|....|....|....|....|....|
PA01    CAGCGGGTGCTGGAGATCCTCTACCTCAAGTGCGAACGCAGCGACGAGAACGAGCCGCTGTTGCGCCGCCGCGAGCTGCTCGAGAAGCAGGGGCAACGCT
MEX001  ....................................................................................................
MEX002  ............................................................C.......................................
MEX003  ............................................................C.......................................
MEX004  ....................................................................................................
MEX005  ....................................................................................................
MEX006  .................................................................T..................................
MEX007  ....................................................................................................
MEX008  ....................................................................................................
MEX009  ....................................................................................................
MEX010  ....................................................................................................
MEX011  ....................................................................................................
MEX012  ............................................................C.......................................
MEX013  ....................................................................................................
MEX014  ....................................................................................................
MEX015  ........................T.T.........................................................................
MEX016  ....................................................................................................
MEX017  ....................................................................................................
MEX018  ....................................................................................................
MEX019  ............................-.......................................................................
MEX020  ....................................................................................................
MEX021  ....................................................................................................
MEX022  ....................................................................................................
MEX023  ....................................................................................................
MEX024  ........................-...........................................................................
MEX025  ...................C................................................................................

                    510       520       530       540       550       560       570       580       590       600
        ....|....|....|....|....|....|....|....|....|....|....|....|....|....|....|....|....|....|....|....|
PA01    TCGGCCTCCGGCAGATCCGCCGGGCGGTGGAACGCGGCGAACTGCCGGCGCGGCTGGACGTCGAGCTGGCC-------AGCATCTATCTGCAATCGCTCT
MEX001  ...............................G.......................................-------......................
MEX002  ...............................G.......................................-------......................
MEX003  ...............................G.......................................-------......................
MEX004  ...............................G.......................................-------......................
MEX005  ...............A...............G.......................................-------......................
MEX006  ...............................G.........T.............................-------......................
```

```
MEX007  ...............................G.......................................-------......................
MEX008  ...............................G.......................................-------......................
MEX009  ...............................G.......................................-------......................
MEX010  ........T......................G.......................................-------......................
MEX011  ...............................G.......................................-------......................
MEX012  ...............................G.......................................-------......................
MEX013  ...............................G.......................................AGCATCC......................
MEX014  ...............................G.......................................-------......................
MEX015  ...............................G.......................................-------......................
MEX016  ...............................G.......................................-------......................
MEX017  .............................----------------------------------------------------------.............
MEX018  ...............................G.......................................-------......................
MEX019  ...............................G.......................................-------......................
MEX020  ...............................G.......................................-------......................
MEX021  ...............................G.......................................-------......................
MEX022  ...............................G.......................................-------......................
MEX023  ...............................G.......................................-------......................
MEX024  ...............................G.......................................-------......................
MEX025  ...............................G.......................................-------......................

                 610       620       630       640       650       660       670       680       690       700
        ....|....|....|....|....|....|....|....|....|....|....|....|....|....|....|....|....|....|....|....|
PA01    GGGACGGCATCTGCGGCACCCTGGCCTGGACCGAGCGCCTGCGCGACGATCCCTGGAACCGCGC----CGAACGCATGTTCCGCGCCGGCCTCGACAGCC
MEX001  ...........................................-----................----................................
MEX002  ................................................................----................................
MEX003  ................................................................----................................
MEX004  ................................................................GCGC................................
MEX005  ................................................................----................................
MEX006  ................................................................----................................
MEX007  ................................................................----................................
MEX008  ................................................................GC--................................
MEX009  ................................................................----................................
MEX010  ..............----------........................................----................................
MEX011  ................................................................----................................
MEX012  ................................................................----................................
MEX013  ................................................................----................................
MEX014  ................................................................----................................
MEX015  ................................................................----................................
MEX016  ..........................................................T.....----................................
MEX017  ................................................................----................................
MEX018  ....----------..................................................----................................
MEX019  ................................................................----................................
MEX020  ...........................................................G--------------..........................
MEX021  ................................................................----................................
MEX022  ...........................................................---------------------....................
MEX023  ................................................................----................................
MEX024  ................................................................----................................
MEX025  ................................................................----................................

                 710       720       730       740       750       760       770
        ....|....|....|....|....|....|....|....|....|....|....|....|....|....|...
PA01    TGCGCAGTTCTCCCTACCTGTTGCTGGCGGACGCCTGAGGGCGTCAATCGTCCGCCATCAGGTGCCTGCGCTG
MEX001  .........................................................................
MEX002  .........................................................................
MEX003  .........................................................................
MEX004  .........................................................................
MEX005  .........................................................................
MEX006  .........................................................................
MEX007  .........................................................................
MEX008  .........................................................................
MEX009  .........................................................................
MEX010  .........................................................................
MEX011  .........................................................................
MEX012  .........................................................................
MEX013  .........................................................................
MEX014  .........................................................................
MEX015  .........................................................................
MEX016  ...................................--------------........................
MEX017  .........................................................................
MEX018  .........................................................................
MEX019  .........................................................................
MEX020  .........................................................................
MEX021  .........................................................................
MEX022  .........................................................................
MEX023  .........................................................................
MEX024  .........................................................................
MEX025  .........................................................................
```

Supplementary Figure 1. Nucleotide sequence variation among the 25 *Pseudomonas aeruginosa mexZ* gene sequences.
