# Supplementary material for: Genotypic Diversity within a Single Pseudomonas aeruginosa Strain Commonly Shared by Australian Patients with Cystic Fibrosis
Source: PLoS One. 2015 Dec 3;10(12):e0144022. doi: 10.1371/journal.pone.0144022 (PMC4669131; doi:10.1371/journal.pone.0144022)
Supplement: S2 Fig — (PDF) [file pone.0144022.s002.pdf]

```
                 10        20        30        40        50        60        70        80        90       100
         ....|....|....|....|....|....|....|....|....|....|....|....|....|....|....|....|....|....|....|....|
PA01     MALVDGFLELERSSGKLEWSAILQKMASDLGFSK---ILFGL--LPKDSQDYENAFIVGNYP--AAWREHYDRAGYARVDPTVS--HCTQS--VLPIFWE
LASR001  ..................................---.....--..................--....................--.....--.......
LASR002  ..................................---.....--..................--....................--.....--.......
LASR003  ..................................---.....--..................--....................--.....--.......
LASR004  ..................................---.....--..................---.ITTGLATR.ST.RSV..PRAY.RF.GNRPSTRR.
LASR005  ..................................---.....--..................--....................--.R...--.......
LASR006  ..................................---.....--..................--....................--.....--.......
LASR007  ..................................---.....--..................--....................--.....--.......
LASR008  ..................................---.....--..................--....................--.....--.......
LASR009  ..................................---.....--..................--....................--.....--.......
LASR010  ..................................---.....--..................--....................--.....--.......
LASR011  ..................R...............---.....--..................--....................--.....--.......
LASR012  ................................LEDPVRPVAG--Q.GLRERLHRRQLP.RLA--R.L.PGWL...RPDGQSLYP--ER.AD--F.GTVHL
LASR013  ..................................---.....--..................--....................--.....--.......
LASR014  ..................................---.....--..................--....................--.....--.......
LASR015  ..................................---.....--..................--....................--.....--.......
LASR016  ..................................---.....--..................--....................--.....--.......
LASR017  ..................................---.....--..................--....................--.....--.......
LASR018  ..................................---.....--..................--....................--.....--.......
LASR019  ..................................---.....--..................--....................--.....--.......
LASR020  ..................................---.....--..................--....................--.....--.......
LASR021  ..................................---.....--..................--....................--.....--.......
LASR022  ..................................-----ILG--Q.GLRERLHRRQLP.RLA--R.L.PGWL...RPDGQSLYP--ER.AD--F.GTVHL
LASR023  ..................................---.....--..................--...............N....--.....--.......
LASR024  ..................................---.....--..................--....................--.....--.......
LASR025  ..................................---.....CC.RTARTTRTPSSSATTR.PG.SITTGLATR.ST.RSV..PRAY.RF.GNRPSTRR.
LASR026  ..................................---.....--..................--....................--.....--.......
LASR027  ..................................---.....--..................--....................--.....--.......
LASR028  ..................................---.....--..................--....................--.....--.......
LASR029  ..................................---.....--..................--....................--.....--.......
LASR030  ..................................---.....--..................--....................--.....--.......
LASR031  ..................................---.....--..........V.......--...............G....--.....--.......
LASR032  ..................................---.....--..................--....................--.....--.......
LASR033  ..................................---.....--..................--....................--.....--.......
LASR034  .......................-..........---.....--..................--....................--.....--.......
LASR035  ..................................---.....--..................--....................--.....--.......
LASR036  ..................................---.....--..................--....................--.....--.......
LASR037  ..................................---.....--..................--..C-................--.....--.......
LASR038  ..................................---.....--..................--....................--.....--.......
LASR039  ..................................---.....--..................--....................--.....--.......
LASR040  ..................................---.....--..................--....................--.....--.......
```

```
                  110       120       130       140       150       160       170       180       190       200
              ....|....|....|....|....|....|....|....|....|....|....|....|....|....|....|....|....|....|....|....|
PA01          PSIYQTRKQHEFFEEASAAG------LVYGLTMPLHGARGELGALSLSVEAENRAEAN---RFMESVLPTLWMLKDYALQSGAGLAFEHPVSKPVVLTSR
LASR001       ....................------................................---.......................................
LASR002       ....................------.....I..........................---.......................................
LASR003       ....................------................................---.......................................
LASR004       S.TSSS..PRP---P.WCM.------PCRCMVLAANS..----.SAWKRKTGP.PTVS----WSR.CR.CGCSRTTHCRAVPDWPS--NIR.ANRWFPAG
LASR005       ....................------................................---.......................................
LASR006       ....................------................................---.......................................
LASR007       ....................------................................R--FMES.CR.CGCSRTTHCRAVPDWPS--NIR.ANRWFPAG
LASR008       ....................R-----PCRCMVLAANS..----.SAWKRKTGP.PTVS----WSR.CR.CGCSRTTHCRAVPDWPS--NIR.ANRWFPAG
LASR009       ....................------................................RFMESVLPT.WM.KDYALQSGGT.LRTSGQQTGGSD---QPG
LASR010       ......-.............------................................---.......................................
LASR011       ....................------................................---.......................................
LASR012       .DAKAA.VLRGSLGRR--P.------VWADHAAAWCSR.TRRAEPQRGSGKPG.GQPFHGVGPADP.DAQGLRTAERCR-T.LRTSGQQTGGSD---QPG
LASR013       ................----------SAA..VYGRCSR.TRRAEPQRGSGKPG.GQPFHGVGPADP.DAQGLRTAERCR-T.LRTSGQQTGGSD---QPG
LASR014       ....................LVY-GGPCRCMVLAANS..----.SAWKRKTGP.PTVS----WSR.CR.CGCSRTTHCRAVPDWPS--NIR.ANRWFPAG
LASR015       ....................------................................---.......................................
LASR016       ....................------................................---.......................................
LASR017       ....................------................................---.......................................
LASR018       ....................------................................---.......................................
LASR019       ....................------................................---.......................................
LASR020       ....................------................................---.......................................
LASR021       ....................------................................---.......................................
LASR022       .DAKAA.VLRGSLGRR--P.------VWADHAAAWCSR.TRRAEPQRGSGKPG.GQPFHGVGPADP.DAQGLRTAERCR-T.LRTSGQQTGGSD---QPG
LASR023       ....................------................................---.......................................
LASR024       ....................------..........................---...---.......................................
LASR025       S.TSSS..PRP---P.WCM.------PCRCMVLAANS..----.SAWKRKTGP.PTVS----WSR.CR.CGCSRTTHCRAVPDWPS--NIR.ANRWFPAG
LASR026       ....................------................................R-FMDADP.DAQGLRTAERCR-T.LRTSGQQTGGSD---QPG
LASR027       ....................------................................---.......................................
LASR028       ....................LVYGLTMPRCMVLAANS..----.SAWKRKTGP.PTVS----WSR.CR.CGCSRTTHCRAVPDWPS--NIR.ANRWFPAG
LASR029       ....................------................................---.............................R.ANRWFPAG
LASR030       .............-......------................................---.......................................
LASR031       ....................------................................---.......................................
LASR032       ....................------................................---......................................W
LASR033       ....................------................................---.......................................
LASR034       ....................------................................---.......................................
LASR035       ....................------................................---.......................................
LASR036       ....................------................................---.......................................
LASR037       ....................------................................---.......................................
LASR038       .................-.A------VWADHAAAWCSR.TRRAEPQRGSGKPG.GQPFHGVGPADP.DAQGLRTAERCR-T.LRTSGQQTGGSD---QPG
LASR039       ....................------................................---.......................................
LASR040       ....................------................................---.......................................
```

```
                  210       220       230       240       250
         ....|....|....|....|....|....|....|....|....|....|....|....
PA01     EKEVLQWCAIGKTSWEISVICNCSEANVNFHMGNIRRKFGVTSRRVAAIMAVNLGLITL
LASR001  ...........................................................
LASR002  ...........................................................
LASR003  ...........................................C...............
LASR004  RR---KC.SGAPSARPVGRYR--LS.TARKP.TS.WEI..GS.VPP..RPLWP.IWVL.
LASR005  ...........................................................
LASR006  ..................................................G........
LASR007  RR---KC.SGAPSARPVGRYR--LS.TARKP.TS.WEI..GS.VPP..RPLWP.IWVL.
LASR008  RR---KC.SGAPSARPVGRYR--LS.TARKP.TS.WEI..GS.VPP..RPLWP.IWVL.
LASR009  .GS.AVVRHRQDQLGD.GYLQ-LLGSQCELPY.KYSAEVRCDLPPRSGHYGRFGSYYS.
LASR010  ...........................................................
LASR011  ...........................................................
LASR012  .GS.AVVRHRQDQLGD.GYLQ-LLGSQCELPY.KYSAEVRCDLPPRSGHYGRFGSYYS-
LASR013  .GS.AVVRHRQDQLGD.GYLQ-LLGSQCELPY.KYSAEVRCDLPPRSGHYGRFGSYYS.
LASR014  RR---KC.SGAPSARPVGRYR--LS.TARKP.TS.WEI..GS.VPP..RPLWP.IWVL.
LASR015  .....................................N.....................
LASR016  ...............................................V..G........
LASR017  .......................................V...................
LASR018  ..............-............................................
LASR019  ..---EVLQWCAIGKTSWRYR--LS.TARKP.TS.WEI..GS.VPP..RPLWP.IWVL.
LASR020  .................................................HYGRF.SYYS
LASR021  ..............................R............................
LASR022  .GS.AVVRHRQDQLGD.GYLQ-LLGSQCELPY.KYSAEVRCDLPPRSGHYGRFGSYYS-
LASR023  ...........................................................
LASR024  ...........................................................
LASR025  RR---KC.SGAPSARPVGRYR--LS.TARKP.TS.WEI..GS.VPP..RPLWP.IWVL.
LASR026  .GS.AVVRHRQDQLGD.GYLQ-LLGSQCELPY.KYSAEVRCDLPPRSGHYGRFGSYYS.
LASR027  ..............................................SGHYGRFGSYYS-
LASR028  RR---KC.SGAPSARPVGRYR--LS.TARKP.TS.WEI..GS.VPP..RPLWP.IWVL.
LASR029  RR---KC.SGAPSARPVGRYR--LS.TARKP.TS.WEI..GS.VPP..RPLWP.IWVL.
LASR030  ...........................................................
LASR031  ...........................................................
LASR032  ...........................................................
LASR033  .................................................YGRFGSYYS-
LASR034  ...........................................................
LASR035  ....................Y......................................
LASR036  ....................................W......................
LASR037  ...........................................................
LASR038  .GS.AVVRHRQDQLGD.GYLQ-LLGSQCELPY.KYSAEVRCDLPPRSGHYGRFGSYYS.
LASR039  ..................................................V........
LASR040  ............A..............................................
```

Supplementary Figure 2. Sequence variation among the 40 *Pseudomonas aeruginosa* LasR amino acid sequences.
