# Supplementary material for: Genotypic Diversity within a Single Pseudomonas aeruginosa Strain Commonly Shared by Australian Patients with Cystic Fibrosis
Source: PLoS One. 2015 Dec 3;10(12):e0144022. doi: 10.1371/journal.pone.0144022 (PMC4669131; doi:10.1371/journal.pone.0144022)
Supplement: S1 Table — (DOCX) [file pone.0144022.s003.docx]

| **S1 Table.** Intra-strain diversity of the *mexZ* and LasR sequences among the 190 *Pseudomonas aeruginosa* isolates | | | | | |
| --- | --- | --- | --- | --- | --- |
| ***mexZ/lasR* subtype** | ***P. aeruginosa* strain^a^** | **No. of isolates** | **No. of patients^b^** | **Year of isolation**  **(No. of isolates)** | **Hospital^c^**  **(No. of isolates)** |
| M8L1 | AUST-01 | 4 | 2 | 2002 (1); 2007-2008 (3) | TPCH (4) |
| M11L1 | AUST-01 | 3 | 3 | 2002 (2); 2007 (1) | RCH (1); TPCH (2) |
| M11L17 | AUST-01 | 1 | 1 | 2008 (1) | TPCH (1) |
| M11L19 | AUST-01 | 1 | 1 | 2007 (1) | TPCH (1) |
| M13L1 | AUST-01 | 1 | 1 | 2007 (1) | TPCH (1) |
| M17L1 | AUST-01 | 3 | 2 | 2002 (2); 2008 (1) | RCH (1); TPCH (2) |
| M17L13 | AUST-01 | 1 | 1 | 2007 (1) | TPCH (1) |
| M23L1 | AUST-01 | 1 | 1 | 2007 (1) | TPCH (1) |
| M24L1 | AUST-01 | 1 | 1 | 2008 (1) | TPCH (1) |
| M25L18 | AUST-01 | 1 | 1 | 2007 (1) | TPCH (1) |
| M2L1 | AUST-02 | 73 | 47 | 2001-2004 (35); 2007-2008 (38) | RCH (27); TPCH (46) |
| M2L3 | AUST-02 | 7 | 4 | 2002 (4); 2007 (3) | RCH (1); TPCH (6) |
| M2L20 | AUST-02 | 4 | 3 | 2002 (1); 2007 (3) | RCH (1); TPCH (3) |
| M2L26 | AUST-02 | 7 | 2 | 2002 (2); 2007 (5) | TPCH (7) |
| M2L28 | AUST-02 | 3 | 2 | 2007 (3) | TPCH (3) |
| M2L30 | AUST-02 | 2 | 2 | 2007 (2) | RCH (1); TPCH (1) |
| M2L11 | AUST-02 | 1 | 1 | 2007 (1) | TPCH (1) |
| M2L12 | AUST-02 | 1 | 1 | 2001 (1) | TPCH (1) |
| M2L16 | AUST-02 | 1 | 1 | 2007 (1) | TPCH (1) |
| M2L18 | AUST-02 | 2 | 1 | 2007 (2) | TPCH (2) |
| M2L2 | AUST-02 | 1 | 1 | 2007 (1) | TPCH (1) |
| M2L21 | AUST-02 | 1 | 1 | 2001 (1) | TPCH (1) |
| M2L22 | AUST-02 | 1 | 1 | 2002 (1) | TPCH (1) |
| M2L23 | AUST-02 | 3 | 1 | 2007 (3) | TPCH (3) |
| M2L24 | AUST-02 | 4 | 1 | 2002 (1); 2007 (3) | TPCH (4) |
| M2L25 | AUST-02 | 1 | 1 | 2002 (1) | TPCH (1) |
| M2L27 | AUST-02 | 1 | 1 | 2008 (1) | TPCH (1) |
| M2L29 | AUST-02 | 1 | 1 | 2002 (1) | RCH (1) |
| M2L31 | AUST-02 | 1 | 1 | 2002 (1) | RCH (1) |
| M2L32 | AUST-02 | 2 | 1 | 2007 (2) | TPCH (2) |
| M2L33 | AUST-02 | 1 | 1 | 2007 (1) | RCH (1) |
| M2L34 | AUST-02 | 1 | 1 | 2002 (1) | RCH (1) |
| M2L35 | AUST-02 | 1 | 1 | 2007 (1) | RCH (1) |
| M2L36 | AUST-02 | 1 | 1 | 2002 (1) | RCH (1) |
| M2L37 | AUST-02 | 2 | 1 | 2007 (2) | RCH (2) |
| M2L38 | AUST-02 | 1 | 1 | 2001 (1) | TPCH (1) |
| M2L4 | AUST-02 | 1 | 1 | 2007 (1) | TPCH (1) |
| M2L40 | AUST-02 | 1 | 1 | 2002 (1) | TPCH (1) |
| M2L5 | AUST-02 | 1 | 1 | 2007 (1) | TPCH (1) |
| M2L6 | AUST-02 | 1 | 1 | 2007 (1) | TPCH (1) |
| M2L8 | AUST-02 | 2 | 1 | 2007 (2) | TPCH (2) |
| M3L39 | AUST-02 | 1 | 1 | 2002 (1) | TPCH (1) |
| M3L1 | AUST-02 | 2 | 1 | 2001 (1); 2007 (1) | TPCH (2) |
| M3L7 | AUST-02 | 16 | 10 | 2007 (16) | TPCH (16) |
| M3L32 | AUST-02 | 2 | 1 | 2001 (2) | TPCH (2) |
| M5L1 | AUST-06 | 4 | 2 | 2002 (2); 2007 (2) | RCH (2); TPCH (2) |
| M5L9 | AUST-06 | 1 | 1 | 2007 (1) | TPCH (1) |
| M22L1 | AUST-11 | 1 | 1 | 2002 (1) | RCH (1) |
| M1L1 | UNIQUE | 1 | 1 | 2007 (1) | TPCH (1) |
| *Continued* | | | | | |
| **S1 Table.** *Continued* | | | | | |
| M4L1 | UNIQUE | 2 | 2 | 2002-3 (2) | 402 (2) |
| M6L1 | UNIQUE | 1 | 1 | 2007 (1) | 403 (1) |
| M7L10 | UNIQUE | 1 | 1 | 2002 (1) | 402 (1) |
| M9L1 | UNIQUE | 1 | 1 | 2002 (1) | 403 (1) |
| M10L1 | UNIQUE | 1 | 1 | 2002 (1) | 403 (1) |
| M12L1 | UNIQUE | 2 | 1 | 2002 (1); 2007 (1) | 403 (2) |
| M14L1 | UNIQUE | 1 | 1 | 2001 (1) | 403 (1) |
| M15L1 | UNIQUE | 1 | 1 | 2002 (1) | 402 (1) |
| M16L1 | UNIQUE | 1 | 1 | 2001 (1) | 403 (1) |
| M18L1 | UNIQUE | 1 | 1 | 2008 (1) | 403 (1) |
| M19L14 | UNIQUE | 1 | 1 | 2002 (1) | 402 (1) |
| M20L15 | UNIQUE | 1 | 1 | 2007 (1) | 403 (1) |
| M21L2 | UNIQUE | 1 | 1 | 2007 (1) | 403 (1) |

^a^ *P. aeruginosa* strain as determined by ERIC-PCR and Sequenom iPlex SNP-based genotyping.

^b^ 55/105(52%) patients contributed more than one *P. aeruginosa* isolate. Of these 55 patients, 18 had isolates with more than two *mexZ*/LasR subtypes. Thus, the column total exceeds the numbers of patients providing specimens.

^c^ TPCH, The Prince Charles Hospital; RCH, Royal Children’s Hospital
