# Supplementary material for: Genotypic Diversity within a Single Pseudomonas aeruginosa Strain Commonly Shared by Australian Patients with Cystic Fibrosis
Source: PLoS One. 2015 Dec 3;10(12):e0144022. doi: 10.1371/journal.pone.0144022 (PMC4669131; doi:10.1371/journal.pone.0144022)
Supplement: S2 Table — (DOCX) [file pone.0144022.s004.docx]

**Supplementary Table 2: Primer sets used in *mexZ* and *lasR* sequencing**

| **Target Gene** | **Sequence** | **Reference** |
| --- | --- | --- |
| ***lasR-F*** | TCCGTGCCCGGCAGGCCTGA | This study |
| ***lasR-R*** | GGCAGTCGTTTCGAGAATGG | This study |
| ***mexZ-F*** | TCGTGAACTCGCGAGCTTG | Morita et al^1^ |
| ***mexZ-R*** | CACATCAGCGAGGAAGACGC | Morita et al^1^ |
| **AUST-02-F** | GCGCCTTCGGCCAGACT | This study |
| **AUST-02-R** | CTGGCGTTTTCGTCGGGTA | This study |

**Reference**

1. Morita Y, Sobel ML, Poole K. Antibiotic inducibility of the MexXY multidrug efflux system of *Pseudomonas aeruginosa:* involvement of the antibiotic-inducible PA5471 gene product. *J Bacteriol* 2006; **188**: 1847-55.
