# Supplementary material for: Genotypic Diversity within a Single Pseudomonas aeruginosa Strain Commonly Shared by Australian Patients with Cystic Fibrosis
Source: PLoS One. 2015 Dec 3;10(12):e0144022. doi: 10.1371/journal.pone.0144022 (PMC4669131; doi:10.1371/journal.pone.0144022)
Supplement: S3 Table — (DOCX) [file pone.0144022.s005.docx]

| **S3 Table.** Minimal inhibitory concentrations (mg/L), number of non-susceptible antibiotic results and multidrug resistance among the 74 AUST-02 strain isolates^a,b^ | | | | | | | | | | | | | | | | | | | | | | | |
| --- | --- | --- | --- | --- | --- | --- | --- | --- | --- | --- | --- | --- | --- | --- | --- | --- | --- | --- | --- | --- | --- | --- | --- |
| **Isolate** | ***mexZ/*LasR subtype** | | **MEM** | | | | **IMI** | | **CAZ** | | **TIM** | | **AZT** | | **COL** | | **CIP** | | **TOB** | | **No. of NS-antibiotics*** | | **MDR** |
| 1 | M2L1 | | 16 (R) | | | | 64 (R) | | 32 (R) | | 64/2 (R) | | 16 (I) | | 4 (I) | | 4 (R) | | 32 (R) | | 8 | | + |
| 2 | M2L1 | | 2 (S) | | | | 4 (I) | | 128 (R) | | 128/2 (R) | | 4 (S) | | 4 (I) | | 4 (R) | | 128 (R) | | 5 | | + |
| 3 | M2L1 | | 8 (R) | | | | 16 (R) | | 16 (I) | | 32/2 (R) | | 16 (I) | | 4 (I) | | 4 (R) | | 4 (S) | | 7 | | + |
| 4 | M2L1 | | 1 (S) | | | | 1 (S) | | 4 (S) | | 16/2 (I) | | 128 (R) | | 4 (I) | | 1 (S) | | 1 (S) | | 3 | | - |
| 5 | M2L1 | | ≤0.25 (S) | | | | 2 (S) | | 1 (S) | | 41672 (S) | | ≤0.25 (S) | | 4 (I) | | 0.5 (S) | | 2 (S) | | 1 | | - |
| 6 | M2L1 | | 8 (R) | | | | 32 (R) | | 64 (R) | | 64/2 (R) | | 128 (R) | | 8 (R) | | 8 (R) | | ≥256 (R) | | 8 | | + |
| 7 | M2L1 | | 2 (S) | | | | 2 (S) | | ≥256 (R) | | 16/2 (I) | | 2 (S) | | 2 (S) | | 2 (I) | | 8 (I) | | 4 | | + |
| 8 | M2L1 | | 8 (R) | | | | 8 (R) | | 128 (R) | | 32/2 (R) | | 8 (S) | | ≤0.25 (S) | | 4 (R) | | 8 (I) | | 5 | | + |
| 9 | M2L1 | | 8 (R) | | | | 32 (R) | | 4 (S) | | 1/2 (S) | | 2 (S) | | 1 (S) | | 8 (R) | | 8 (I) | | 3 | | - |
| 10 | M2L1 | | 0.5 (S) | | | | 1 (S) | | 32 (R) | | 16/2 (I) | | 128 (R) | | 1 (S) | | 0.5 (S) | | 8 (I) | | 2 | | - |
| 11 | M2L1 | | 2 (S) | | | | 1 (S) | | 128 (R) | | 64/2 (R) | | 4 (S) | | ≤0.25 (S) | | 0.5 (S) | | 8 (I) | | 3 | | - |
| 12 | M2L1 | | 0.5 (S) | | | | 1 (S) | | 64 (R) | | 64/2 (R) | | 16 (I) | | 1 (S) | | 2 (I) | | ≥256 (R) | | 5 | | + |
| 13 | M2L1 | | 4 (I) | | | | 16 (R) | | 64 (R) | | 64/2 (R) | | 16 (I) | | 2 (S) | | 4 (R) | | 16 (R) | | 7 | | + |
| 14 | M2L1 | | 32 (R) | | | | 32 (R) | | 64 (R) | | 128/2 (R) | | 32 (R) | | 2 (S) | | 4 (R) | | ≥256 (R) | | 7 | | + |
| 15 | M2L1 | | ≤0.25 (S) | | | | 2 (S) | | 128 (R) | | 4/2 (S) | | 64 (R) | | ≤0.5 (S) | | 8 (R) | | 16 (R) | | 4 | | + |
| 16 | M2L1 | | 8 (R) | | | | 4 (I) | | 16 (I) | | 16/2 (I) | | 8 (S) | | 1 (S) | | 4 (R) | | 128 (R) | | 6 | | - |
| 17 | M2L1 | | 16 (R) | | | | 32 (R) | | 4 (S) | | 32/2 (R) | | 2 (S) | | 2 (S) | | 4 (R) | | 128 (R) | | 5 | | + |
| 18 | M2L1 | | 32 (R) | | | | 64 (R) | | 16 (I) | | 8/2 (S) | | 8 (S) | | 2 (S) | | 8 (R) | | 32 (R) | | 5 | | + |
| 19 | M2L1 | | 16 (R) | | | | 32 (R) | | 16 (I) | | 8/2 (S) | | 8 (S) | | ≤0.25 (S) | | 2 (I) | | 4 (S) | | 4 | | - |
| 20 | M2L1 | | 16 (R) | | | | 16 (R) | | 32 (R) | | 32/2 (R) | | 64 (R) | | 1 (S) | | 4 (R) | | 4 (S) | | 6 | | + |
| 21 | M2L1 | | 16 (R) | | | | 16 (R) | | 8 (S) | | 64/2 (R) | | 4 (S) | | 2 (S) | | 4 (R) | | 4 (S) | | 4 | | - |
| 22 | M2L1 | | 8 (R) | | | | 32 (R) | | 8 (S) | | 64/2 (R) | | 4 (S) | | 2 (S) | | 8 (R) | | 4 (S) | | 4 | | - |
| 23 | M2L1 | | 2 (S) | | | | 128 (R) | | 64 (R) | | 8/2 (S) | | 1 (S) | | 1 (S) | | 4 (R) | | 4 (S) | | 3 | | - |
| 24 | M2L1 | | 1 (S) | | | | 8 (R) | | 16 (I) | | 2/2 (S) | | 1 (S) | | 2 (S) | | 0.5 (S) | | 1 (S) | | 2 | | - |
| 25 | M2L1 | | ≤0.25 (S) | | | | 2 (S) | | 64 (R) | | 2/2 (S) | | 1 (S) | | ≤0.25 (S) | | 1 (S) | | 1 (S) | | 1 | | - |
| *Continued* | | | | | | | | | | | | | | | | | | | | | | | |
| **S3 Table.** *Continued* | | | | | | | | | | | | | | | | | | | | | | | |
| 26 | M2L3 | | 1 (S) | | | | 2 (S) | | 8 (S) | | 64/2 (R) | | 2 (S) | | 2 (S) | | 0.5 (S) | | ≥256 (R) | | 2 | | - |
| 27 | | M2L4 | | 2 (S) | | 2 (S) | | 64 (R) | | 32/2 (R) | | 32 (R) | | 2 (S) | | 2 (I) | | ≥256 (R) | | 5 | | + | |
| 28 | | M2L5 | | 16 (R) | | 32 (R) | | 64 (R) | | 128/2 (R) | | 128 (R) | | ≤0.25 (S) | | 8 (R) | | 8 (I) | | 7 | | + | |
| 29 | | M2L6 | | 2 (S) | | 4 (I) | | 128 (R) | | 2/2 (S) | | 64 (R) | | ≤0.25 (S) | | 0.5 (S) | | 2 (S) | | 3 | | - | |
| 30 | | M2L8 | | 8 (R) | | 4 (I) | | 128 (R) | | 64/2 (R) | | 64 (R) | | 0.5 (S) | | ≤0.25 (S) | | 16 (R) | | 5 | | - | |
| 31 | | M2L8 | | 16 (R) | | 16 (R) | | 128 (R) | | 64/2 (R) | | 64 (R) | | ≤0.25 (S) | | 1 (S) | | 16 (R) | | 6 | | + | |
| 32 | | M2L8 | | 32 (R) | | 8 (R) | | 128 (R) | | 64/2 (R) | | 128 (R) | | 0.5 (S) | | ≤0.25 (S) | | 32 (R) | | 6 | | + | |
| 33 | | M2L12 | | 64 (R) | | 16 (R) | | 32 (R) | | 64/2 (R) | | 128 (R) | | 2 (S) | | 4 (R) | | 4 (S) | | 6 | | - | |
| 34 | | M2L20 | | 8 (R) | | 8 (R) | | 16 (I) | | 4/2 (S) | | 2 (S) | | 2 (S) | | 2 (I) | | 8 (I) | | 5 | | + | |
| 35 | | M2L23 | | 4 (I) | | 8 (R) | | 128 (R) | | 64/2 (R) | | ≥256 (R) | | 4 (I) | | 2 (I) | | 4 (S) | | 7 | | + | |
| 36 | | M2L24 | | 0.25 (S) | | 2 (S) | | 8 (S) | | 16/2 (I) | | 4 (S) | | 1 (S) | | 1 (S) | | ≥256 (R) | | 2 | | - | |
| 37 | | M2L24 | | 0.5 (S) | | 2 (S) | | 16 (I) | | 8/2 (S) | | 4 (S) | | 1 (S) | | 1 (S) | | ≥256 (R) | | 2 | | - | |
| 38 | | M2L24 | | 2 (S) | | 4 (I) | | 32 (R) | | 64/2 (R) | | 16 (I) | | 1 (S) | | 2 (I) | | ≥256 (R) | | 6 | | + | |
| 39 | | M2L24 | | 4 (I) | | 4 (I) | | 32 (R) | | 64/2 (R) | | 16 (I) | | 1 (S) | | 1 (S) | | ≥256 (R) | | 6 | | + | |
| 40 | | M2L25 | | 4 (I) | | 2 (S) | | 16 (I) | | 64/2 (R) | | 8 (S) | | 1 (S) | | 1 (S) | | 4 (S) | | 3 | | - | |
| 41 | | M2L25 | | 8 (R) | | 16 (R) | | 8 (S) | | 64/2 (R) | | 4 (S) | | 2 (S) | | 1 (S) | | ≥256 (R) | | 4 | | - | |
| 42 | | M2L25 | | 16 (R) | | 64 (R) | | 8 (S) | | 32/2 (R) | | 8 (S) | | ≤0.5 (S) | | 2 (I) | | ≥256 (R) | | 4 | | + | |
| 43 | | M2L25 | | 32 (R) | | 64 (R) | | 32 (R) | | 64/2 (R) | | 16 (I) | | 2 (S) | | 4 (R) | | ≥256 (R) | | 6 | | + | |
| 44 | | M2L26 | | 0.5 (S) | | 4 (I) | | 4 (S) | | ≤0.25/2 (S) | | 2 (S) | | 1 (S) | | ≤0.25 (S) | | 1 (S) | | 1 | | - | |
| 45 | | M2L26 | | 1 (S) | | 8 (R) | | 4 (S) | | ≤0.25/2 (S) | | 4 (S) | | 2 (S) | | 1 (S) | | 4 (S) | | 1 | | - | |
| 46 | | M2L26 | | 2 (S) | | 16 (R) | | 8 (S) | | ≤0.25/2 (S) | | 2 (S) | | 2 (S) | | ≤0.25 (S) | | ≥256 (R) | | 2 | | - | |
| 47 | | M2L26 | | 4 (I) | | 4 (I) | | 8 (S) | | ≤0.25/2 (S) | | 1 (S) | | 2 (S) | | 1 (S) | | 8 (I) | | 3 | | - | |
| 48 | | M2L26 | | 4 (I) | | 4 (I) | | 8 (S) | | ≤0.25/2 (S) | | 2 (S) | | 2 (S) | | 1 (S) | | 8 (I) | | 3 | | - | |
| 49 | | M2L26 | | 1 (S) | | 2 (S) | | 64 (R) | | 128/2 (R) | | 8 (S) | | ≤0.5 (S) | | 1 (S) | | ≥256 (R) | | 3 | | - | |
| 50 | | M2L26 | | 1 (S) | | 16 (R) | | 32 (R) | | ≤0.25/2 (S) | | 8 (S) | | 2 (S) | | ≤0.25 (S) | | 256 (R) | | 3 | | - | |
| 51 | | M2L26 | | 4 (I) | | 16 (R) | | 8 (S) | | 4/2 (S) | | 2 (S) | | 2 (S) | | ≤0.25 (S) | | ≥256 (R) | | 3 | | - | |
| 52 | | M2L28 | | 0.5 (S) | | 16 (R) | | 128 (R) | | 32/2 (R) | | 128 (R) | | 256 (R) | | 2 (I) | | ≥256 (R) | | 7 | | + | |
| 53 | | M2L30 | | 32 (R) | | 32 (R) | | 8 (S) | | 32/2 (R) | | 8 (S) | | 2 (S) | | 4 (R) | | 64 (R) | | 5 | | + | |
| *Continued* | | | | | | | | | | | | | | | | | | | | | | | |
| **S3 Table .** *Continued* | | | | | | | | | | | | | | | | | | | | | | | |
| 54 | | M2L36 | | 4 (I) | | 4 (I) | | 128 (R) | | ≥256/2 (R) | | 128 (R) | | 2 (S) | | 0.5 (S) | | 32 (R) | | 6 | | + | |
| 55 | | M3L1 | | ≤0.25 (S) | | 4 (I) | | 8 (S) | | ≤0.25/2 (S) | | 1 (S) | | ≤0.25 (S) | | 2 (I) | | 2 (S) | | 2 | | - | |
| 56 | | M3L1 | | | 0.5 (S) | 4 (I) | | 32 (R) | | 64/2 (R) | | 1 (S) | | 256 (R) | | 2 (I) | | 16 (R) | | 6 | | + | |
| 57 | | M3L7 | | | 32 (R) | 32 (R) | | 128 (R) | | 128/2 (R) | | 128 (R) | | 4 (I) | | 16 (R) | | 4 (S) | | 8 | | + | |
| 58 | | M3L7 | | | 32 (R) | 64 (R) | | 128 (R) | | 256/2 (R) | | ≥256 (R) | | 4 (I) | | 16 (R) | | 8 (I) | | 8 | | + | |
| 59 | | M3L7 | | | 16 (R) | 16 (R) | | 64 (R) | | ≥256/2 (R) | | 128 (R) | | 4 (I) | | 4 (R) | | 8 (I) | | 8 | | + | |
| 60 | | M3L7 | | | 32 (R) | 32 (R) | | 128 (R) | | 256/2 (R) | | ≥256 (R) | | 4 (I) | | 4 (R) | | 8 (I) | | 8 | | + | |
| 61 | | M3L7 | | | 32 (R) | 64 (R) | | 64 (R) | | 256/2 (R) | | ≥256 (R) | | 16 (R) | | 4 (R) | | 16 (R) | | 8 | | + | |
| 62 | | M3L7 | | | 8 (R) | 16 (R) | | 64 (R) | | 256/2 (R) | | ≥256 (R) | | 64 (R) | | 2 (I) | | 16 (R) | | 8 | | + | |
| 63 | | M3L7 | | | 64 (R) | 64 (R) | | 64 (R) | | 256/2 (R) | | ≥256 (R) | | 8 (R) | | 1 (S) | | 8 (I) | | 7 | | + | |
| 64 | | M3L7 | | | 8 (R) | 32 (R) | | 128 (R) | | 128/2 (R) | | 32 (R) | | 8 (R) | | 4 (R) | | 8 (I) | | 8 | | + | |
| 65 | | M3L7 | | | 16 (R) | 32 (R) | | 32 (R) | | ≥256/2 (R) | | 32 (R) | | 16 (R) | | 4 (R) | | 8 (I) | | 8 | | + | |
| 66 | | M3L7 | | | 16 (R) | 32 (R) | | 64 (R) | | ≥256/2 (R) | | 128 (R) | | 8 (R) | | 4 (R) | | 8 (I) | | 8 | | + | |
| 67 | | M3L7 | | | 32 (R) | 64 (R) | | 64 (R) | | 256/2 (R) | | ≥256 (R) | | 8 (R) | | 4 (R) | | 8 (I) | | 8 | | + | |
| 68 | | M3L7 | | | 16 (R) | 32 (R) | | 32 (R) | | 256/2 (R) | | 16 (I) | | 8 (R) | | 4 (R) | | 8 (I) | | 8 | | + | |
| 69 | | M3L7 | | | 32 (R) | 64 (R) | | 128 (R) | | 256/2 (R) | | ≥256 (R) | | 2 (S) | | 4 (R) | | 4 (S) | | 6 | | + | |
| 70 | | M3L7 | | | 16 (R) | 32 (R) | | 64 (R) | | ≥256/2 (R) | | 64 (R) | | 2 (S) | | 4 (R) | | 16 (R) | | 7 | | + | |
| 71 | | M3L7 | | | 8 (R) | 16 (R) | | ≥256 (R) | | ≥256/2 (R) | | ≥256 (R) | | 1 (S) | | 4 (R) | | 16 (R) | | 7 | | + | |
| 72 | | M3L7 | | | 16 (R) | 32 (R) | | 64 (R) | | ≥256/2 (R) | | 64 (R) | | 1 (S) | | 2 (I) | | 16 (R) | | 8 | | + | |
| 73 | | M3L32 | | | ≤0.25 (S) | 1 (S) | | 4 (S) | | 1/2 (S) | | 1 (S) | | ≤0.25 (S) | | 2 (I) | | 4 (S) | | 1 | | - | |
| 74 | | M3L32 | | | ≤0.25 (S) | 1 (S) | | 128 (R) | | 1/2 (S) | | 1 (S) | | ≤0.25 (S) | | 2 (I) | | 4 (S) | | 2 | | - | |

^a^ Abbreviations: MEM, meropenem; IMI, imipenem; CAZ, ceftazidime; AZT, aztreonam; TIM, ticarcillin/clavulanate; COL, colistin sulphate; CIP, ciprofloxacin; TOB, tobramycin; NS, non-susceptible; MDR, multidrug resistance as defined by resistance to all antibiotics tested in at least two of the three antibiotic classes: aminoglycosides (tobramycin), fluoroquinolone (ciprofloxacin) and beta-lactams (ceftazidime, meropenem, imipenem, ticarcillin-clavulanate, and aztreonam).

^b^ Minimal inhibitory concentrations were determined using broth microdilution according to Clinical Laboratory and Standards Institute 2013 guidelines and breakpoints.^20^

^c^ NS-antibiotics = non-susceptible antibiotics defined as either ‘resistant’ or ‘intermediate’ on broth microdilution MIC testing.
